# Enhancing breast cancer treatment selection through 2TLIV*q*-ROFS-based multi-attribute group decision making

**DOI:** 10.3389/frai.2024.1402719

**Published:** 2024-06-03

**Authors:** Muhammad Waheed Rasheed, Abid Mahboob, Anfal Nabeel Mustafa, Israa Badi, Zainab Abdulkhaleq Ahmed Ali, Zainb H. Feza

**Affiliations:** ^1^Department of Mathematics, Division of Science and Technology, University of Education, Lahore, Pakistan; ^2^Department of Pharmacy, Al-Noor University College, Nineveh, Iraq; ^3^College of Pharmacy, National University of Science and Technology, Nasiriyah, Iraq; ^4^College of MLT, University of Ahl Al Bayt, Karbala, Iraq; ^5^Department of Pharmacy, Al-Zahrawi University College, Karbala, Iraq

**Keywords:** MAGDM, MABAC method, 2TLIV*q*-ROFS, 2TLIV*q*-ROFWA operator, 2TLIV*q*-ROFWJ operator, breast cancer treatment selection

## Abstract

**Introduction:**

Breast cancer is an extremely common and potentially fatal illness that impacts millions of women worldwide. Multiple criteria and inclinations must be taken into account when selecting the optimal treatment option for each patient.

**Methods:**

The selection of breast cancer treatments can be modeled as a multi-attribute group decision-making (MAGDM) problem, in which a group of experts evaluate and rank alternative treatments based on multiple attributes. MAGDM methods can aid in enhancing the quality and efficacy of breast cancer treatment selection decisions. For this purpose, we introduce the concept of a 2-tuple linguistic interval-valued *q*-rung orthopair fuzzy set (2TLIV*q*-ROFS), a new development in fuzzy set theory that incorporates the characteristics of interval-valued *q*-rung orthopair fuzzy set (IV*q*-ROFS) and 2-tuple linguistic terms. It can express the quantitative and qualitative aspects of uncertain information, as well as the decision-makers' level of satisfaction and dissatisfaction.

**Results:**

Then, the 2TLIV*q*-ROF weighted average (2TLIV*q*-ROFWA) operator and the 2TLIV*q*-ROF weighted geometric (2TLIV*q*-ROFWJ) operator are introduced as two new aggregation operators. In addition, the multi-attribute border approximation area comparison (MABAC) method is extended to solve the MAGDM problem with 2TLIV*q*-ROF information.

**Discussion:**

To demonstrate the efficacy and applicability of the suggested model, a case study of selecting the optimal breast cancer treatment is presented. The results of the computations show that the suggested MAGDM model is able to handle imprecision and subjectivity in complicated decision-making scenarios and opens new research scenarios for scholars.

## 1 Introduction

Treating breast cancer needs different kinds of treatment from different medical experts. Khajehkhasan and Fakheri (2020) evaluated mammographic images of patients' breasts with the help of doctors. Shastri et al. (2020) found how tumors turned into malignant or benign with the help of machine learning techniques. Several factors, including the stage and subtype of cancer, the patient's age and general health, the patient's preferences and values, and the availability and accessibility of diverse treatment options, influence the selection of the most suitable treatment for each individual. Among the most common treatments for breast cancer are surgery, radiation therapy, chemotherapy, hormone therapy, targeted therapy, and immunotherapy. Each of these treatments has its own benefits and risks, and they can be used individually or in combination with others. The goal of cancer treatment is to get rid of cancer cells, delay their return, and improve the patient's quality of life and chances of survival. The breast cancer treatment decision-making process entails a dialogue between the patient and the healthcare team, which may include oncologists, surgeons, radiologists, pathologists, nurses, social workers, and other specialists. The patient should be informed of the diagnosis, prognosis, treatment options, potential outcomes, adverse effects, and cost of each option. In addition, the patient should be encouraged to ask inquiries, express concerns and preferences, and seek a second opinion when necessary. Respecting and upholding the patient's autonomy and values throughout the treatment journey is the responsibility of the healthcare team. The most prevalent cancer in the globe, breast cancer is a global public health problem. Survival rates and patient wellbeing are increased by effective treatment plans. However, selecting a treatment for breast cancer is a complex and challenging utilization involving numerous considerations, alternatives, and stakeholders, consequently, it is a MAGDM issue. MAGDM is a branch of decision science that focuses on situations where multiple decision-makers (DMs) evaluate multiple alternatives based on multiple criteria. In the context of breast cancer treatment selection, alternatives refer to the available treatment options, including surgery, radiation therapy, endocrine therapy, chemotherapy, and immunotherapy. The criteria are the variables that influence the treatment outcomes, including survival rate, toxicity, cost, quality of life, and patient preference. The stakeholders are the individuals or groups who are involved in or affected by the treatment decision, such as patients, doctors, nurses, family members, and insurance companies. To address a MAGDM problem, various methods and tools can be utilized to aggregate the preferences and opinions of the stakeholders and to rank or select the optimal alternative(s) based on the attribute. Incorporating evidence-based data, expert knowledge, and patient values, this paper applies the MABAC method to 2TLIV*q*-ROFS information in order to provide clinical decision support for breast cancer treatment selection. Table 1 represents the summary of existing research on breast cancer treatment selection.

**Table 1:** Summary of existing research on breast cancer treatment selection.

**References**	**Research methodology**	**Key finding**
Camgoz Akdag and Menekse (2023)	Spherical fuzzy CRITIC-REGIME	Planning for breast cancer therapy
Hernández-Julio et al. (2023)	Intelligent fuzzy system	Predict the breast cancer dataset from Wisconsin
Chatterjee and Das (2023a)	Fuzzy linguistic multi-criteria decision-making system	Breast cancer diagnosis and grading
Chatterjee and Das (2023b)	Integrating probabilistic fuzzy logic and multilayer perceptron	Diagnostic and staging treatments for breast cancer
Lin et al. (2023)	Vector deep fuzzy neural network	Categorization of breast cancer
Krasnov et al. (2023)	Fuzzy c-means clustering	Breast cancer screening technology
Gupta et al. (2023)	Fuzzy rule-based system with decision tree	Cancer diagnosis for breasts
Ghorbani et al. (2023)	Fuzzy-PSO	Breast tumor detection
Khan et al. (2023)	Computer vision and artificial intelligence techniques	Forecasting breast cancer
Vidivelli and Devi (2023)	Fuzzy entropy segmentation and ensemble classification	Mechanism to detecting breast cancer
Singh et al. (2023)	Soft computing approach	The categorization of cancer of the breast
Sravanthi et al. (2023)	Enhanced LSTM framework STOA-based preference for features	IoT-based breast cancer detection and therapy
Mohanty and Champati (2023)	A robust classification model using fuzzy logic	Accurate prognosis of breast cancer patients
Balaji et al. (2023)	Optimized segmentation scheme and supervised algorithm	Breast cancer diagnosis using a diagnostic system that is computerized

Multi-attribute decision making (MADM) refers to making preference decisions by evaluating and prioritizing a limited set of alternatives based on multiple conflict attributes (Nafei et al., 2021, 2023; Akram et al., 2023d; Farajpour Khanaposhtani, 2023). Soltanifar (2024) presented a hybrid method based on a linear programming model for solving MADM problems by combining two new methods, the COPRAS and the MOORA and also using the concept of discrimination intensity functions. Shakerian et al. (2023) selected the best contractor in one of the projects of dairy companies in Fars province, after holding a tender and completing a questionnaire by project experts, using AHP and VIKOR methods. In the field of decision sciences, MAGDM holds significant importance as it provides a fascinating framework for analyzing and resolving complex problems. In the modern era, decision-making is used in digital transformation for a more informed and effective approach (Haudi, 2024; Jones, 2024; Min and Kim, 2024). It examines how to encourage a panel of DMs in differing preferences and viewpoints to reach a consensus on a complex problem involving multiple criteria and options. MAGDM can be applied to numerous domains, including project management, resource allocation, environmental planning, social choice, and more. MAGDM methods typically involve four steps: defining the problem structure, soliciting the individual preferences, aggregating the preferences into a group preference, and choosing the best alternative(s) based on the group preference. There are numerous challenges and opportunities in MAGDM research, including how to handle uncertainty, inconsistency, and conflict among DMs, how to design effective and efficient preference induction and aggregation mechanisms, how to incorporate human factors and ethical considerations, and how to evaluate and compare various MAGDM methods. Regarding the circumstances of real-world MADM, intuitionistic fuzzy set (IFS) was conceptualized by Atanassov (1986) in this discussion, represent a valuable extension of fuzzy set (FS) (Zadeh, 1965). IFS is characterized by its capacity to assign each element both a membership degree (MD) and a non-membership degree (NMD), with the combined values' sum cannot be more than 1.

Rasoulzadeh et al. (2022) proposed a new combined Markowitz and the cross data envelopment analysis models utilizing the IF numbers. However, in practical decision-making scenarios, it can occur that the squared sum of an alternative's MD and NMD, as per the DMs criteria, exceeds 1. This presents a challenge for IFS but is effectively addressed by Pythagorean fuzzy set (PyFS) (Yager, 2013). Ismail et al. (2023) proposed the incorporation of a Bonferroni mean aggregation operator within a Pythagorean neutrosophic environment, illustrated through a numerical example applied to DEMATEL. PyFS ensures that the squared sum of their MD and NMD remains equal to or less than 1, as illustrated by example: A support for membership of DM in an alternative is $\frac{\sqrt{3}}{3}$, and his support against membership is ½. The sum of these values is indeed >1, underscoring the inadequacy of IFS to handle this situation. In contrast, PyFS, with ${\left(\frac{\sqrt{3}}{3}\right)}^{2}+{\left(\frac{1}{2}\right)}^{2}\leq 1$, competently capture such ambiguity. Evidently, PyFS is better suited to model ambiguity in real-world MADM problems compared to IFS. Furthermore, development of *q*-rung orthopair fuzzy sets (*q*-ROFS) (Yager, 2016) has gained recognition as a valuable approach to capturing ambiguity in MADM situations. *q*-ROFS distinguish from other existing FSs due to its MD and NMD characteristics, where the total of the *q*th powers of MD and NMD does not exceed 1. For instance, when (0.8+0.1) ≤ 1, it represents an IFS and when (0.7 + 0.5)^2^ ≤ 1, it denotes a PyFS, though it is not considered an IFS when the MD is 0.5. This scenario cannot be effectively described using either IFS or PyFS if the NMD is 0.8. In this case, (0.8, 0.7) represents a *q*-ROF number (*q* = 3), and the *q*-ROFS proves to be the suitable approach to address this situation. IFS and PyFS, both falling under the category of *q*-ROFS, shows the generality of *q*-ROFS. As the rung *q* increases, the scope of permissible orthopairs expands that adhere to the bounding constraint. This feature empowers *q*-ROFS to represent a wider range of fuzzy information. In essence, the diversity of data expression can be flexibly determined by varying the parameter *q*, making *q*-ROFS exceptionally adaptable and well-suited for handling uncertainty in various environments.

However, while *q*-ROFS has been utilized in numerous applications of MAGDM, its limitations are becoming increasingly evident. There are instances where DMs face challenges quantifying their judgments with precise numerical values due to incomplete information. In such scenarios, it becomes more practical for DMs to convey their assessments using a subset of the closed interval [0, 1]. In response to this need, Joshi et al. (2018) introduced IV*q*-ROFS, where membership and non-membership degrees are represented as intervals instead of single real numbers. This approach also involved the investigation of operations like negation, union, intersection, and set operations. Wan et al. (2023) devised an innovative integrated group decision-making method for evaluating the quality of systems using IV*q*-ROFS, particularly in the context of selecting the best software product from multiple alternatives. Gurmani et al. (2023) aimed to establish a novel methodology for determining expert weights through distance and similarity measures by using IV*q*-ROFNs. Luqman and Shahzadi (2023) developed the IV*q*-ROF superiority and inferiority technique, incorporating sine-trigonometric operational laws. Xu (2023) proposed a two-stage MADM method that used the IV*q*-ROF technique to address complex problems, such as the selection of a bike-sharing recycling supplier. Combining complex IV*q*-ROF information with linguistic sets, Qi et al. (2023) introduced the concept of complex interval-valued *q*-rung orthopair linguistic information, which is more generalized and exceptionally practical for representing challenging and unreliable information in complex situations. Ahemad et al. (2023) developed the COPRAS approach to address MAGDM problems using IV*q*-ROFNs.

It can be challenging to quantitatively present judgements in decision-making at times. Zadeh (1975) made a distinction between linguistic and numerical data to get around this problem. He introduced the idea that linguistic terms can be used to describe qualitative information and established the concept of linguistic variables. 2TL preference relations are useful instruments for resolving situations in which DMs are likely to employ linguistic variables to represent data for evaluation. To more clearly describe the qualitative information in MADM situations through linguistic terms, Herrera and Martínez (2000) suggested a new model called the 2TL representation model. The 2TL Fermatean FS is a useful tool that brings together the benefits of the reliable 2TL model and Fermatean FS. Akram et al. (2023a) set out to create new decision-making methods based on 2TL Fermatean FS that could deal with circumstances where linguistic labels were applied to specific data. Akram et al. (2023b) introduced a novel 2TLPyF-MULTIMOORA approach to solving the undergraduate teaching audit and evaluation problem, which depends on the the 2TLPyFS and the MULTIMOORA technique. Akram et al. (2023c) proposed and utilized the Dombi operations to develop certain aggregation operators: 2TL *q*-rung picture fuzzy Dombi weighted averaging operator and 2TL *q*-rung picture fuzzy Dombi weighted geometric operator. By combining cloud theory and rough approximations with a 2TL situation, Sarwar (2023) suggested a novel mathematical model. First, three novel language manipulation models, 2TL clouds, 2TL rough numbers, and Dual 2TL rough number clouds, were developed to handle unpredictability with randomness and multi-granularity at the same time. Jin et al. (2023) proposed a 2TL decision-making technique that coupled a consistency adjustment algorithm with a 2TL data envelopment analysis model in order to maintain the initial preference information of DMs.

In 2015, Pamučar and Ćirović (2015) proposed the MABAC method as a MADM technique. This method looks at different options by seeing how close they are to the best and worst values within a certain area called the border approximation area (BAA). Therefore, MABAC can manage conflicting attributes and provides a more precise ranking result than other MADM methods such as RAFSI, MAIRCA, VIKOR, or MARCOS. Demir et al. (2024) presented a comprehensive bibliometric analysis of MABAC method using the Biblioshiny application of the bibliometrix package, R program and VOSviewer tools to provide a holistic view of the research landscape by identifying its evolution, major contributors and most influential research areas. In light of numerous options and ambiguous, subjective, and unreliable data, Shang et al. (2023) sought to identify the optimal MADM method. The modified rough *Z*-number MABAC approach used Pugh's controlled convergence, rough number, *Z*-number, consistency theory, and Shannon entropy. Ran (2023) proposed the maximizing deviation method for determining the attribute weights for the single-valued neutrosophic set (SVNS), and the SVNN-MABAC method was designed for MAGDM under SVNS to evaluate the lending performance of sustainable microfinance organization. With the goal to establish the feasibility of the suggested methodology, Jana et al. (2023) presented a novel method to construct MABAC approach utilizing PyF numbers. To determine the most effective plastic waste management procedure in situations where the attribute weights and decision expert weights are completely unknown, Mandal and Seikh (2023) developed a hybrid MAGDM methodology combining the entropy method, deviation-based method, and MABAC method with interval-valued spherical FS. Given that risk assessment is complex and uncertain, and that cognitive fuzziness and psychological behavior of experts further complicate matters, combining prospect theory and the MABAC method in a fuzzy Fermatean environment, Tan et al. (2023) developed an integrated MAGDM risk investment evaluation framework. The modified MABAC method with *Z*-numbers was utilized by Božanic et al. (2023) to identify the most effective contingency strategy for managing oil release risks. Zorlu and Dede (2023) used CRITIC method to analyze criteria weights, while MABAC established their priority values for alternatives.

### 1.1 Motivation

Millions of women throughout the world are affected by the common and fatal disease known as breast cancer. To increase patient outcomes and survival rates, it is crucial to improve the decision-making process for choosing a course of treatment. It is difficult for doctors and DMs to choose a breast cancer treatment because there are different criteria and preferences. This hard subject can be approached methodically and completely using a structured MAGDM framework. The uncertainty and confusion in deciding on breast cancer treatment can make it hard for traditional methods to handle it well. By introducing the 2TLIV*q*-ROFS approach, assessments can be made more flexible and exact, which improves the representation of expert viewpoints. By ensuring that the final treatment choice is well-founded and takes into account all pertinent information, the development of novel aggregation operators further improves the precision of the decision-making process. By expanding the applicability of this well-known strategy and enabling it to be handled to the unique issues of breast cancer treatment selection, the MABAC method can be extended to the 2TLIV*q*-ROFS environment to make better decisions. Doctors and DMs can confidently use this strategy because the suggested model has shown it works well and is practical. Moreover, the sensitivity analysis can prove that the proposed method is wide and reliable. The proposed strategy can outperform over other techniques and contribute to better decision-making in the selection of breast cancer treatment by a comparative analysis with existing methodologies.

### 1.2 Objectives

The research study is done in order to accomplish the following objectives:

- The challenge of selecting the best treatment option for breast cancer patients is addressed by proposing a MAGDM framework.
- The concept of 2TLIV*q*-ROFS is introduced as a novel way for DMs to present their evaluations within a wider scope as well as improve their ability to handle uncertain knowledge more efficiently.
- A variety of novel aggregation operators are established, including the 2TLIV*q*-ROFWA and the 2TLIV*q*-ROFWJ operators, to improve the precision of current aggregation techniques while considering the interconnection of the 2TLIV*q*-ROF numbers (2TLIV*q*-ROFNs).
- The MABAC method is extended to handle the MAGDM problem under 2TLIV*q*-ROFS information, enabling better decision-making in breast cancer treatment selection.
- A case study of breast cancer treatment selection is demonstrated which shows the efficacy and practicability of proposed model in handling imprecision and subjectivity in complex decision-making environments.
- The resilience and adaptability of the proposed model is assessed by performing a sensitivity analysis to determine how the parameter *q* affects decision-making.
- Comparative analysis with existing decision-making approaches is done which shows that it improves breast cancer treatment selection.

### 1.3 Structure

Organization of this article is as follows: Section 2 discusses the definitions of 2TL representation model and IV*q*-ROFS. Section 3 introduces the 2TLIV*q*-ROFS, a new data representation concept. Additionally, this part discusses operational principles, the score function, and the accuracy function of 2TLIV*q*-ROFNs. Section 4 discusses the average and geometric operators with their weighted forms for 2TLIV*q*-ROFNs, as well as their properties. Section 5 examines the 2TLIV*q*-ROF-MABAC method for MAGDM problem-solving. Section 6 gives a breast cancer therapy case study to demonstrate the viability and effectiveness of the suggested methodology. Section 7 provide insights, acknowledge limitations, and suggest future study directions.

### 1.4 Nomenclature

Nomenclature of the proposed work is shown in Table 2.

**Table 2:** Nomenclature of the proposed work.

**Notation**	**Description**
2TLIV*q*-ROFS	2-tuple linguistic interval-valued *q*-rung orthopair fuzzy set
2TLIV*q*-ROFWA	2TLIV*q*-ROF weighted average
2TLIV*q*-ROFWG	2TLIV*q*-ROF weighted geometric
MABAC	Multi-attribute border approximation area comparison
𝔏 = {𝕁_ℓ_|ℓ = 1, …, τ}	Linguistic term set
$R=\{\left(\left[\left({𝕁}_{𝔯}\left(\varrho \right),ℜ\left(\varrho \right)\right),\left({𝕁}_{𝔱}\left(\varrho \right),𝔗\left(\varrho \right)\right)\right]\right)$	2TL*q*-ROFN
$\left([({𝕁}_{𝔲}\left(\varrho \right),𝔘\left(\varrho \right)),\left({𝕁}_{y}\left(\varrho \right),Y\left(\varrho \right)\right)])\right\}$	
([(𝕁_𝔯_(ϱ), ℜ(ϱ)), (𝕁_𝔱_(ϱ), 𝔗(ϱ))])	Membership degree of 2TLIV*q*-ROFN
$\left(\left[\left({𝕁}_{𝔲}\left(\varrho \right),𝔘\left(\varrho \right)\right),\left({𝕁}_{y}\left(\varrho \right),Y\left(\varrho \right)\right)\right]\right)$	Non-membership degree of 2TLIV*q*-ROFN
Γ	Alternative
Θ	Attribute
K(R)	Score function of 2TLIV*q*-ROFN
F(R)	Accuracy function of 2TLIV*q*-ROFN

## 2 Preliminaries

In this section, we will explore key concepts related to our research study such as 2TL terms and IV*q*-ROFS.

Definition 1. Herrera and Martínez (2000) Let 𝔏 = {𝕁_ℓ_|ℓ = 1, …, τ} be a linguistic term set (LTS), and ξ ∈ [0, τ] indicate the linguistic symbolic aggregation outcome. Description of the function Δ to acquire 2TL information identical to ξ:


$$\begin{array}{l} \Delta :\left[0,\tau \right]\to 𝔏\times \left[-0.5,0.5\right),\\ \Delta \left(\xi \right)=\{\begin{array}{l} {𝕁}_{\ell },\ell =\text{round}\left(\xi \right),\\ \upsilon =\xi -\ell ,\;\upsilon \in \left[-0.5,0.5\right). \end{array} \end{array}$$

Definition 2. Herrera and Martínez (2000) Let 𝔏 = {𝕁_ℓ_|ℓ = 1, …, τ} be a LTS and (𝕁_ℓ_, υ_ℓ_) being a 2-tuple, then there is a function Δ^−1^ that converts the 2-tuple to its analogous number ξ ∈ [0, τ]⊂*R*, where


$$\begin{array}{r} \Delta^{-1}:𝔏\times \left[-0.5,0.5\right)\to \left[0,\tau \right],\\ \Delta^{-1}\left({𝕁}_{\ell },\upsilon \right)=\ell +\upsilon =\xi . \end{array}$$

Definition 3. Joshi et al. (2018) An IV*q*-ROFS A derived on the basis of the fixed set P can be described as in the following Equation 1.


(1) $$\begin{array}{l} A=\left\{\varrho ,\mu_{A}\left(\varrho \right),\nu_{A}\left(\varrho \right)|\varrho \in P\right\} \end{array}$$

where $\mu_{A}\left(\varrho \right)$ and $\nu_{A}\left(\varrho \right)$ are two intervals, denoted as [*u, v*] and [*x, y*], respectively, representing the MD and NMD of the element ϱ∈P with respect to the set A. These intervals meet the requirement $0\leq \text{Sup}{\left(\mu_{A}\left(\varrho \right)\right)}^{q}+\text{Sup}{\left(\nu_{A}\left(\varrho \right)\right)}^{q}\leq 1$ for all ϱ∈P. To achieve simplicity, we indicate to this pair of intervals as an IV*q*-ROFN, represented as ℜ = ([*u, v*], [*x, y*]).

## 3 2-Tuple linguistic interval-valued *q*-rung orthopair fuzzy set

In the field of FS theory, the 2TLIV*q*-ROFS is a novel development. This section illustrates the structure and operational laws of the proposed 2TLIV*q*-ROFS. Combining the concepts of 2TL terms and IV*q*-ROFS resulted in the innovative idea of 2TLIV*q*-ROFS. Considering that the *q*th power of MD as well as NMD is included, the proposed set is more flexible.

Definition 4. Assume 𝔏 = {𝕁_ℓ_|ℓ = 0, 1, …, τ} indicates a LTS that has an odd cardinality. If ([(𝕁_𝔯_(ϱ), ℜ(ϱ)), (𝕁_𝔱_(ϱ), 𝔗(ϱ))], $[({𝕁}_{𝔲}(\varrho ),𝔘(\varrho )),({𝕁}_{y}(\varrho ),Y(\varrho ))])$ is defined for ${𝕁}_{𝔯}\left(\varrho \right),{𝕁}_{𝔱}\left(\varrho \right),{𝕁}_{𝔲}\left(\varrho \right),{𝕁}_{y}\left(\varrho \right),\in 𝔏,ℜ\left(\varrho \right),𝔗\left(\varrho \right),𝔘\left(\varrho \right),Y\left(\varrho \right)\in \left[-0.5,0.5\right)$, where ([(𝕁_𝔯_(ϱ), ℜ(ϱ)), (𝕁_𝔱_(ϱ), 𝔗(ϱ))]) and $\left(\left[\left({𝕁}_{𝔲}\left(\varrho \right),𝔘\left(\varrho \right)\right),\left({𝕁}_{y}\left(\varrho \right),Y\left(\varrho \right)\right)\right]\right)$ indicate the MD and NMD with respective 2TL terms. The 2TLIV*q*-ROFS is characterized by the following Equation 2:


(2) $$\begin{array}{l} ℛ=\{\varrho ,([({𝕁}_{r}(\varrho ),ℜ(\varrho )),({𝕁}_{t}(\varrho ),T(\varrho ))],[({𝕁}_{u}(\varrho ),U(\varrho )),\\ ({𝕁}_{y}(\varrho ),Y(\varrho ))])|\varrho \in P\}, \end{array}$$

where $\Delta^{-1}\left({𝕁}_{𝔯}\left(\varrho \right),ℜ\left(\varrho \right)\right)$, $\Delta^{-1}\left({𝕁}_{𝔱}\left(\varrho \right),𝔗\left(\varrho \right)\right)$, $\Delta^{-1}\left({𝕁}_{𝔲}\left(\varrho \right),𝔘\left(\varrho \right)\right)$, $\Delta^{-1}\left({𝕁}_{y}\left(\varrho \right),Y\left(\varrho \right)\right)\in \left[0,\tau \right]$ and $0\leq \text{Sup}(\Delta^{-1}[({𝕁}_{𝔯}\left(\varrho \right),ℜ\left(\varrho \right)),$ $\left({𝕁}_{𝔱}\left(\varrho \right),𝔗\left(\varrho \right))\right]{)}^{q}+\text{Sup}{(\Delta^{-1}[({𝕁}_{𝔲}\left(\varrho \right),𝔘\left(\varrho \right)),\left({𝕁}_{y}\left(\varrho \right),Y\left(\varrho \right)\right)])}^{q}\leq \tau^{q}.$

In order to compare any two 2TLIV*q*-ROFNs, their score and accuracy functions are described in the following Equations 3 and 4:

Definition 5. Let $R=\left(\left[\left({𝕁}_{𝔯},ℜ\right),\left({𝕁}_{𝔱},𝔗\right)\right],\left[\left({𝕁}_{𝔲},𝔘\right),\left({𝕁}_{y},Y\right)\right]\right)$ be a 2TLIV*q*-ROFN. Afterward, the score function K and the accuracy function F are established as:


(3) $$\begin{array}{r} K(ℛ)=\Delta {\left(\frac{\tau }{4}((1+(\frac{\Delta^{-1}({𝕁}_{r},ℜ)}{\tau }\right)}^{q}-{\left(\frac{\Delta^{-1}({𝕁}_{u},U)}{\tau }\right)}^{q})\\ \;+(1+{\left(\frac{\Delta^{-1}({𝕁}_{t},T)}{\tau }\right)}^{q}-{\left(\frac{\Delta^{-1}({𝕁}_{y},Y)}{\tau }\right)}^{q}))),\\ \;\Delta^{-1}(K(ℛ))\in [0,\tau ], \end{array}$$

(4) $$\begin{array}{r} ℱ(ℛ)=\Delta \left(\begin{array}{c} \frac{\tau }{4}{\left(\frac{\Delta^{-1}({𝕁}_{r},ℜ)}{\tau }\right)}^{q}+{\left(\frac{\Delta^{-1}({𝕁}_{t},T)}{\tau }\right)}^{q}\\ +{\left(\frac{\Delta^{-1}({𝕁}_{u},U)}{\tau }\right)}^{q}+{\left(\frac{\Delta^{-1}({𝕁}_{y},Y)}{\tau }\right)}^{q}) \end{array}\right),\\ \Delta^{-1}\left(F\left(R\right)\right)\in \left[0,\tau \right]. \end{array}$$

Definition 6. Let $R_{1}=\left(\left[\left({𝕁}_{{𝔯}_{1}},{ℜ}_{1}\right),\left({𝕁}_{{𝔱}_{1}},{𝔗}_{1}\right)\right],\left[\left({𝕁}_{{𝔲}_{1}},{𝔘}_{1}\right),\left({𝕁}_{y_{1}},Y_{1}\right)\right]\right)$ and $R_{2}=\left(\left[\left({𝕁}_{{𝔯}_{2}},{ℜ}_{2}\right),\left({𝕁}_{{𝔱}_{2}},{𝔗}_{2}\right)\right],\left[\left({𝕁}_{{𝔲}_{2}},{𝔘}_{2}\right),\left({𝕁}_{y_{2}},Y_{2}\right)\right]\right)$ represent two 2TLIV*q*-ROFNs, and subsequently compare both utilizing the aforementioned principles:

(1) If $K\left(R_{1}\right)>K\left(R_{2}\right)$, then $R_{1}>R_{2}$;
(2) If $K\left(R_{1}\right)=K\left(R_{2}\right)$, then
  - If $F\left(R_{1}\right)>F\left(R_{2}\right)$, then $R_{1}>R_{2}$;
  - If $F\left(R_{1}\right)=F\left(R_{2}\right)$, then $R_{1}\sim R_{2}$.

Innovative operational principles of the 2TLIV*q*-ROFNs are described in Definition 7.

Definition 7. Let $R=\left(\left[\left({𝕁}_{𝔯},ℜ\right),\left({𝕁}_{𝔱},𝔗\right)\right],\left[\left({𝕁}_{𝔲},𝔘\right),\left({𝕁}_{y},Y\right)\right]\right)$, $R_{1}=\left(\left[\left({𝕁}_{{𝔯}_{1}},{ℜ}_{1}\right),\left({𝕁}_{{𝔱}_{1}},{𝔗}_{1}\right)\right],\left[\left({𝕁}_{{𝔲}_{1}},{𝔘}_{1}\right),\left({𝕁}_{y_{1}},Y_{1}\right)\right]\right)$ and $R_{2}=\left(\left[\left({𝕁}_{{𝔯}_{2}},{ℜ}_{2}\right),\left({𝕁}_{{𝔱}_{2}},{𝔗}_{2}\right)\right],\left[\left({𝕁}_{{𝔲}_{2}},{𝔘}_{2}\right),\left({𝕁}_{y_{2}},Y_{2}\right)\right]\right)$ are any three 2TLIV*q*-ROFNs and ε be a real positive integer, then

1.
$$\begin{array}{l} R_{1}⊕R_{2}=\left(\begin{array}{l} \left[\begin{array}{l} \Delta \left(\tau \left(\sqrt[{q}]{{\left(\frac{\Delta^{-1}\left({𝕁}_{{𝔯}_{1}},{ℜ}_{1}\right)}{\tau }\right)}^{q}+{\left(\frac{\Delta^{-1}\left({𝕁}_{{𝔯}_{2}},{ℜ}_{2}\right)}{\tau }\right)}^{q}-{\left(\frac{\Delta^{-1}\left({𝕁}_{{𝔯}_{1}},{ℜ}_{1}\right)}{\tau }\right)}^{q}{\left(\frac{\Delta^{-1}\left({𝕁}_{{𝔯}_{2}},{ℜ}_{2}\right)}{\tau }\right)}^{q}}\right)\right),\\ \Delta \left(\tau \left(\sqrt[{q}]{{\left(\frac{\Delta^{-1}\left({𝕁}_{{𝔱}_{1}},{𝔗}_{1}\right)}{\tau }\right)}^{q}+{\left(\frac{\Delta^{-1}\left({𝕁}_{{𝔱}_{2}},{𝔗}_{2}\right)}{\tau }\right)}^{q}-{\left(\frac{\Delta^{-1}\left({𝕁}_{{𝔱}_{1}},{𝔗}_{1}\right)}{\tau }\right)}^{q}{\left(\frac{\Delta^{-1}\left({𝕁}_{{𝔱}_{2}},{𝔗}_{2}\right)}{\tau }\right)}^{q}}\right)\right) \end{array}\right],\\ \left[\begin{array}{l} \Delta \left(\tau \left(\left(\frac{\Delta^{-1}\left({𝕁}_{{𝔲}_{1}},{𝔘}_{1}\right)}{\tau }\right)\left(\frac{\Delta^{-1}\left({𝕁}_{{𝔲}_{2}},{𝔘}_{2}\right)}{\tau }\right)\right)\right),\Delta \left(\tau \left(\left(\frac{\Delta^{-1}\left({𝕁}_{y_{1}},Y_{1}\right)}{\tau }\right)\left(\frac{\Delta^{-1}\left({𝕁}_{y_{2}},Y_{2}\right)}{\tau }\right)\right)\right) \end{array}\right] \end{array}\right). \end{array}$$
2.
$$\begin{array}{l} R_{1}⊗R_{2}=\left(\begin{array}{l} \left[\begin{array}{l} \Delta \left(\tau \left(\left(\frac{\Delta^{-1}\left({𝕁}_{{𝔯}_{1}},{ℜ}_{1}\right)}{\tau }\right)\left(\frac{\Delta^{-1}\left({𝕁}_{{𝔯}_{2}},{ℜ}_{2}\right)}{\tau }\right)\right)\right),\Delta \left(\tau \left(\left(\frac{\Delta^{-1}\left({𝕁}_{{𝔱}_{1}},{𝔗}_{1}\right)}{\tau }\right)\left(\frac{\Delta^{-1}\left({𝕁}_{{𝔱}_{2}},{𝔗}_{2}\right)}{\tau }\right)\right)\right) \end{array}\right],\\ \left[\begin{array}{l} \Delta \left(\tau \left(\sqrt[{q}]{{\left(\frac{\Delta^{-1}\left({𝕁}_{{𝔲}_{1}},{𝔘}_{1}\right)}{\tau }\right)}^{q}+{\left(\frac{\Delta^{-1}\left({𝕁}_{{𝔲}_{2}},{𝔘}_{2}\right)}{\tau }\right)}^{q}-{\left(\frac{\Delta^{-1}\left({𝕁}_{{𝔲}_{1}},{𝔘}_{1}\right)}{\tau }\right)}^{q}{\left(\frac{\Delta^{-1}\left({𝕁}_{{𝔲}_{2}},{𝔘}_{2}\right)}{\tau }\right)}^{q}}\right)\right),\\ \Delta \left(\tau \left(\sqrt[{q}]{{\left(\frac{\Delta^{-1}\left({𝕁}_{y_{1}},Y_{1}\right)}{\tau }\right)}^{q}+{\left(\frac{\Delta^{-1}\left({𝕁}_{y_{2}},Y_{2}\right)}{\tau }\right)}^{q}-{\left(\frac{\Delta^{-1}\left({𝕁}_{y_{1}},Y_{1}\right)}{\tau }\right)}^{q}{\left(\frac{\Delta^{-1}\left({𝕁}_{y_{2}},Y_{2}\right)}{\tau }\right)}^{q}}\right)\right) \end{array}\right] \end{array}\right). \end{array}$$
3.
$$\begin{array}{l} \varepsilon R=\left(\begin{array}{l} \left[\begin{array}{l} \Delta \left(\tau \left(\sqrt[{q}]{1-{\left(1-{\left(\frac{\Delta^{-1}\left({𝕁}_{𝔯},ℜ\right)}{\tau }\right)}^{q}\right)}^{\varepsilon }}\right)\right),\Delta \left(\tau \left(\sqrt[{q}]{1-{\left(1-{\left(\frac{\Delta^{-1}\left({𝕁}_{𝔱},𝔗\right)}{\tau }\right)}^{q}\right)}^{\varepsilon }}\right)\right) \end{array}\right],\\ \left[\begin{array}{l} \Delta \left(\tau \left({\left(\frac{\Delta^{-1}\left({𝕁}_{𝔲},𝔘\right)}{\tau }\right)}^{\varepsilon }\right)\right),\Delta \left(\tau \left({\left(\frac{\Delta^{-1}\left({𝕁}_{y},Y\right)}{\tau }\right)}^{\varepsilon }\right)\right) \end{array}\right] \end{array}\right),\;\varepsilon \geq 0. \end{array}$$
4.
$$\begin{array}{l} R^{\varepsilon }=\left(\begin{array}{l} \left[\begin{array}{l} \Delta \left(\tau \left({\left(\frac{\Delta^{-1}\left({𝕁}_{𝔯},ℜ\right)}{\tau }\right)}^{\varepsilon }\right)\right),\Delta \left(\tau \left({\left(\frac{\Delta^{-1}\left({𝕁}_{𝔱},𝔗\right)}{\tau }\right)}^{\varepsilon }\right)\right) \end{array}\right],\\ \left[\begin{array}{l} \Delta \left(\tau \left(\sqrt[{q}]{1-{\left(1-{\left(\frac{\Delta^{-1}\left({𝕁}_{𝔲},𝔘\right)}{\tau }\right)}^{q}\right)}^{\varepsilon }}\right)\right),\Delta \left(\tau \left(\sqrt[{q}]{1-{\left(1-{\left(\frac{\Delta^{-1}\left({𝕁}_{y},Y\right)}{\tau }\right)}^{q}\right)}^{\varepsilon }}\right)\right) \end{array}\right] \end{array}\right),\;\varepsilon \geq 0. \end{array}$$

Definition 8. Let $R_{1}=\left(\left[\left({𝕁}_{{𝔯}_{1}},{ℜ}_{1}\right),\left({𝕁}_{{𝔱}_{1}},{𝔗}_{1}\right)\right],\left[\left({𝕁}_{{𝔲}_{1}},{𝔘}_{1}\right),\left({𝕁}_{y_{1}},Y_{1}\right)\right]\right)$ and $R_{2}=\left(\left[\left({𝕁}_{{𝔯}_{2}},{ℜ}_{2}\right),\left({𝕁}_{{𝔱}_{2}},{𝔗}_{2}\right)\right],\left[\left({𝕁}_{{𝔲}_{2}},{𝔘}_{2}\right),\left({𝕁}_{y_{2}},Y_{2}\right)\right]\right)$ are two 2TLIV*q*-ROFNs. The 2TLIV*q*-ROF normalized Hamming distance is expressed as follows:


(5) $$\begin{array}{l} d\left(R_{1},R_{2}\right)=\Delta \left(\frac{\tau }{4}\left(\begin{array}{l} \left|{\left(\frac{\Delta^{-1}\left({𝕁}_{{𝔯}_{1}},{ℜ}_{1}\right)}{\tau }\right)}^{q}-{\left(\frac{\Delta^{-1}\left({𝕁}_{{𝔯}_{2}},{ℜ}_{2}\right)}{\tau }\right)}^{q}|+|{\left(\frac{\Delta^{-1}\left({𝕁}_{{𝔱}_{1}},{𝔗}_{1}\right)}{\tau }\right)}^{q}-{\left(\frac{\Delta^{-1}\left({𝕁}_{{𝔱}_{2}},{𝔗}_{2}\right)}{\tau }\right)}^{q}\right|\\ +|{\left(\frac{\Delta^{-1}\left({𝕁}_{{𝔲}_{1}},{𝔘}_{1}\right)}{\tau }\right)}^{q}-{\left(\frac{\Delta^{-1}\left({𝕁}_{{𝔲}_{2}},{𝔘}_{2}\right)}{\tau }\right)}^{q}|+|{\left(\frac{\Delta^{-1}\left({𝕁}_{y_{1}},Y_{1}\right)}{\tau }\right)}^{q}-{\left(\frac{\Delta^{-1}\left({𝕁}_{y_{2}},Y_{2}\right)}{\tau }\right)}^{q}| \end{array}\right)\right), \end{array}$$

where $d\left(R_{1},R_{2}\right)\in \left[0,\tau \right]$.

## 4 The 2TLIV*q*-ROF weighted aggregation operators

The following section describes the 2TLIV*q*-ROFWA and 2TLIV*q*-ROFWJ operators for weighted information aggregation. The two proposed AOs additionally possess the characteristics of idempotency, monotonicity, and boundedness.

Definition 9. Let $R_{ȷ}=([\left({𝕁}_{{𝔯}_{ȷ}},{ℜ}_{ȷ}\right)$, (𝕁_𝔱_ȷ__, 𝔗_ȷ_)], [(𝕁_𝔲_ȷ__, ${𝔘}_{ȷ}),({𝕁}_{y_{ȷ}},Y_{ȷ})])\left(ȷ=1,2,\ldots ,\beta \right)$ be a set of 2TLIV*q*-ROFNs. The 2TLIV*q*-ROFWA operator as represents in Equation 6 the transformation that ensures ⊺^β^ → ⊺ is transformed as follows:


(6) $$\begin{array}{l} \text{2TLIV}q\text{-ROFWA}\left(R_{1},R_{2},\ldots ,R_{\beta }\right)={⊕}_{ȷ=1}^{\beta }\varpi_{ȷ}R_{ȷ}, \end{array}$$

in which ⊺ is the set of 2TLIV*q*-ROFNs, $\varpi ={\left(\varpi_{1},\varpi_{2},\ldots ,\varpi_{\beta }\right)}^{T}$ represents the weight vector of $R_{ȷ}\left(ȷ=1,2,\ldots ,\beta \right)$, satisfies the criteria ϖ_ȷ_ ∈ [0, 1] as well as $\sum_{ȷ=1}^{\beta }\varpi_{ȷ}=1.$

Theorem 1. Consider $R_{ȷ}=\left(\left[\left({𝕁}_{{𝔯}_{ȷ}},{ℜ}_{ȷ}\right),\left({𝕁}_{{𝔱}_{ȷ}},{𝔗}_{ȷ}\right)\right],\left[\left({𝕁}_{{𝔲}_{ȷ}},{𝔘}_{ȷ}\right),\left({𝕁}_{y_{ȷ}},Y_{ȷ}\right)\right]\right)\left(ȷ=1,2,\ldots ,\beta \right)$ be a set of 2TLIV*q*-ROFNs regarding weight vector $\varpi ={\left(\varpi_{1},\varpi_{2},\ldots ,\varpi_{\beta }\right)}^{T}$, in which ϖ_ȷ_ ∈ [0, 1] and $\overset{\beta }{\underset{ȷ=1}{\sum }}\varpi_{ȷ}=1$, then


(7) $$\begin{array}{l} \text{2TLIV}q\text{-ROFWA}\left(R_{1},R_{2},\ldots ,R_{\beta }\right)=\\ \left(\begin{array}{l} \left[\begin{array}{l} \Delta \left(\tau \left(\sqrt[{q}]{1-\overset{\beta }{\underset{ȷ=1}{\prod }}{\left(1-{\left(\frac{\Delta^{-1}\left({𝕁}_{{𝔯}_{ȷ}},{ℜ}_{ȷ}\right)}{\tau }\right)}^{q}\right)}^{\varpi_{ȷ}}}\right)\right),\Delta \left(\tau \left(\sqrt[{q}]{1-\overset{\beta }{\underset{ȷ=1}{\prod }}{\left(1-{\left(\frac{\Delta^{-1}\left({𝕁}_{{𝔱}_{ȷ}},{𝔗}_{ȷ}\right)}{\tau }\right)}^{q}\right)}^{\varpi_{ȷ}}}\right)\right) \end{array}\right],\\ \left[\begin{array}{l} \Delta \left(\tau \overset{\beta }{\underset{ȷ=1}{\prod }}{\left(\frac{\Delta^{-1}\left({𝕁}_{{𝔲}_{ȷ}},{𝔘}_{ȷ}\right)}{\tau }\right)}^{\varpi_{ȷ}}\right),\Delta \left(\tau \overset{\beta }{\underset{ȷ=1}{\prod }}{\left(\frac{\Delta^{-1}\left({𝕁}_{y_{ȷ}},Y_{ȷ}\right)}{\tau }\right)}^{\varpi_{ȷ}}\right) \end{array}\right] \end{array}\right). \end{array}$$

*Proof*. To prove that Equation (8) is valid for positive integer β, we use mathematical induction.

(a) Whenever β = 1, then the subsequent outcome is:
$$\varpi_{1}R_{1}=\left(\begin{array}{l} \left[\begin{array}{l} \Delta \left(\tau \left(\sqrt[{q}]{1-{\left(1-{\left(\frac{\Delta^{-1}\left({𝕁}_{{𝔯}_{1}},{ℜ}_{1}\right)}{\tau }\right)}^{q}\right)}^{\varpi_{1}}}\right)\right),\Delta \left(\tau \left(\sqrt[{q}]{1-{\left(1-{\left(\frac{\Delta^{-1}\left({𝕁}_{{𝔱}_{1}},{𝔗}_{1}\right)}{\tau }\right)}^{q}\right)}^{\varpi_{1}}}\right)\right), \end{array}\right],\\ \left[\begin{array}{l} \Delta \left(\tau {\left(\frac{\Delta^{-1}\left({𝕁}_{{𝔲}_{1}},{𝔘}_{1}\right)}{\tau }\right)}^{\varpi_{1}}\right),\Delta \left(\tau {\left(\frac{\Delta^{-1}\left({𝕁}_{y_{1}},Y_{1}\right)}{\tau }\right)}^{\varpi_{1}}\right) \end{array}\right] \end{array}\right).$$
  Thus, Equation (7) holds for β = 1.
(b) Assume that Equation (7) is valid when β = α,
$$\begin{array}{l} \text{2TLIV}q\text{-ROFWA}\left(R_{1},R_{2},\ldots ,R_{ȷ}\right)\\ =\left(\begin{array}{l} \left[\begin{array}{l} \Delta \left(\tau \left(\sqrt[{q}]{1-\overset{\alpha }{\underset{ȷ=1}{\prod }}{\left(1-{\left(\frac{\Delta^{-1}\left({𝕁}_{{𝔯}_{ȷ}},{ℜ}_{ȷ}\right)}{\tau }\right)}^{q}\right)}^{\varpi_{ȷ}}}\right)\right),\Delta \left(\tau \left(\sqrt[{q}]{1-\overset{\alpha }{\underset{ȷ=1}{\prod }}{\left(1-{\left(\frac{\Delta^{-1}\left({𝕁}_{{𝔱}_{ȷ}},{𝔗}_{ȷ}\right)}{\tau }\right)}^{q}\right)}^{\varpi_{ȷ}}}\right)\right) \end{array}\right],\\ \left[\begin{array}{l} \Delta \left(\tau \overset{\alpha }{\underset{ȷ=1}{\prod }}{\left(\frac{\Delta^{-1}\left({𝕁}_{{𝔲}_{ȷ}},{𝔘}_{ȷ}\right)}{\tau }\right)}^{\varpi_{ȷ}}\right),\Delta \left(\tau \overset{\alpha }{\underset{ȷ=1}{\prod }}{\left(\frac{\Delta^{-1}\left({𝕁}_{y_{ȷ}},Y_{ȷ}\right)}{\tau }\right)}^{\varpi_{ȷ}}\right) \end{array}\right] \end{array}\right). \end{array}$$
  Subsequently, when the value of β = α + 1, we can utilize the inductive assumption to establish the following:
$$\begin{array}{l} \text{2TLIV}q\text{-ROFWA}\left(R_{1},R_{2},\ldots ,R_{\alpha },R_{\alpha +1}\right)\\ =\text{2TLIV}q\text{-ROFWA}\left(R_{1},R_{2},\ldots ,R_{\alpha }\right)⊕\varpi_{\alpha +1}R_{\alpha +1} \end{array}$$
$$\begin{array}{l} =\left(\begin{array}{l} \left[\begin{array}{l} \Delta \left(\tau \left(\sqrt[{q}]{1-\overset{\alpha }{\underset{ȷ=1}{\prod }}{\left(1-{\left(\frac{\Delta^{-1}\left({𝕁}_{{𝔯}_{ȷ}},{ℜ}_{ȷ}\right)}{\tau }\right)}^{q}\right)}^{\varpi_{ȷ}}}\right)\right),\Delta \left(\tau \left(\sqrt[{q}]{1-\overset{\alpha }{\underset{ȷ=1}{\prod }}{\left(1-{\left(\frac{\Delta^{-1}\left({𝕁}_{{𝔱}_{ȷ}},{𝔗}_{ȷ}\right)}{\tau }\right)}^{q}\right)}^{\varpi_{ȷ}}}\right)\right) \end{array}\right],\\ \left[\begin{array}{l} \Delta \left(\tau \overset{\alpha }{\underset{ȷ=1}{\prod }}{\left(\frac{\Delta^{-1}\left({𝕁}_{{𝔲}_{ȷ}},{𝔘}_{ȷ}\right)}{\tau }\right)}^{\varpi_{ȷ}}\right),\Delta \left(\tau \overset{\alpha }{\underset{ȷ=1}{\prod }}{\left(\frac{\Delta^{-1}\left({𝕁}_{y_{ȷ}},Y_{ȷ}\right)}{\tau }\right)}^{\varpi_{ȷ}}\right) \end{array}\right] \end{array}\right)\\ ⊕\left(\begin{array}{l} \left[\begin{array}{l} \Delta \left(\tau \left(\sqrt[{q}]{1-{\left(1-{\left(\frac{\Delta^{-1}\left({𝕁}_{{𝔯}_{\alpha +1}},{ℜ}_{\alpha +1}\right)}{\tau }\right)}^{q}\right)}^{\varpi_{\alpha +1}}}\right)\right),\Delta \left(\tau \left(\sqrt[{q}]{1-{\left(1-{\left(\frac{\Delta^{-1}\left({𝕁}_{{𝔱}_{\alpha +1}},{𝔗}_{\alpha +1}\right)}{\tau }\right)}^{q}\right)}^{\varpi_{\alpha +1}}}\right)\right) \end{array}\right],\\ \left[\begin{array}{l} \Delta \left(\tau {\left(\frac{\Delta^{-1}\left({𝕁}_{{𝔲}_{\alpha +1}},{𝔘}_{\alpha +1}\right)}{\tau }\right)}^{\varpi_{\alpha +1}}\right),\Delta \left(\tau {\left(\frac{\Delta^{-1}\left({𝕁}_{y_{\alpha +1}},Y_{\alpha +1}\right)}{\tau }\right)}^{\varpi_{\alpha +1}}\right) \end{array}\right] \end{array}\right).\\ =\left(\begin{array}{l} \left[\begin{array}{l} \Delta \left(\tau \left(\sqrt[{q}]{1-\overset{\alpha +1}{\underset{ȷ=1}{\prod }}{\left(1-{\left(\frac{\Delta^{-1}\left({𝕁}_{{𝔯}_{ȷ}},{ℜ}_{ȷ}\right)}{\tau }\right)}^{q}\right)}^{\varpi_{ȷ}}}\right)\right),\Delta \left(\tau \left(\sqrt[{q}]{1-\overset{\alpha +1}{\underset{ȷ=1}{\prod }}{\left(1-{\left(\frac{\Delta^{-1}\left({𝕁}_{{𝔱}_{ȷ}},{𝔗}_{ȷ}\right)}{\tau }\right)}^{q}\right)}^{\varpi_{ȷ}}}\right)\right) \end{array}\right],\\ \left[\begin{array}{l} \Delta \left(\tau \overset{\alpha +1}{\underset{ȷ=1}{\prod }}{\left(\frac{\Delta^{-1}\left({𝕁}_{{𝔲}_{ȷ}},{𝔘}_{ȷ}\right)}{\tau }\right)}^{\varpi_{ȷ}}\right),\Delta \left(\tau \overset{\alpha +1}{\underset{ȷ=1}{\prod }}{\left(\frac{\Delta^{-1}\left({𝕁}_{y_{ȷ}},Y_{ȷ}\right)}{\tau }\right)}^{\varpi_{ȷ}}\right) \end{array}\right] \end{array}\right). \end{array}$$

Thus, Equation (7) is valid for a non-negative integer β = α + 1. Therefore, utilizing the method of induction, it is concluded that Equation (7) has validity for any number of β ≥ 1.

Theorem 2. Let $R_{ȷ}=\left(\left[\left({𝕁}_{{𝔯}_{ȷ}},{ℜ}_{ȷ}\right),\left({𝕁}_{{𝔱}_{ȷ}},{𝔗}_{ȷ}\right)\right],\left[\left({𝕁}_{{𝔲}_{ȷ}},{𝔘}_{ȷ}\right),\left({𝕁}_{y_{ȷ}},Y_{ȷ}\right)\right]\right)$ and $R_{ȷ}^{\prime }=\left(\left[\left({𝕁}_{{𝔯}_{ȷ}}^{\prime },{ℜ}_{ȷ}^{\prime }\right),\left({𝕁}_{{𝔱}_{ȷ}}^{\prime },{𝔗}_{ȷ}^{\prime }\right)\right],\left[\left({𝕁}_{{𝔲}_{ȷ}}^{\prime },{𝔘}_{ȷ}^{\prime }\right),\left({𝕁}_{y_{ȷ}}^{\prime },Y_{ȷ}^{\prime }\right)\right]\right)\left(ȷ=1,2,\ldots ,\beta \right)$ are distinct sets of 2TLIV*q*-ROFNs. The 2TLIV*q*-ROFWA operator consequently adheres to the following properties:

1. (Idempotency) When every $R_{ȷ}=\left(\left[\left({𝕁}_{{𝔯}_{ȷ}},{ℜ}_{ȷ}\right),\left({𝕁}_{{𝔱}_{ȷ}},{𝔗}_{ȷ}\right)\right],\left[\left({𝕁}_{{𝔲}_{ȷ}},{𝔘}_{ȷ}\right),\left({𝕁}_{y_{ȷ}},Y_{ȷ}\right)\right]\right)\left(ȷ=1,2,\ldots ,\beta \right)$ is identical with regard to each ȷ, then
$$\begin{array}{l} \text{2TLIV}q\text{-ROFWA}\left(R_{1},R_{2},\ldots ,R_{\beta }\right)=R. \end{array}$$
2. (Monotonicity) If $R_{ȷ}\leq R_{ȷ}^{\prime }$, for all ȷ, then
$$\begin{array}{l} \text{2TLIV}q\text{-ROFWA}\left(R_{1},R_{2},\ldots ,R_{\beta }\right)\leq \\ \text{2TLIV}q\text{-ROFWA}\left(R_{1}^{\prime },R_{2}^{\prime },\ldots ,R_{\beta }^{\prime }\right). \end{array}$$
3. (Boundedness) Let $R_{ȷ}=([({𝕁}_{{𝔯}_{ȷ}}$, ${ℜ}_{ȷ}),({𝕁}_{{𝔱}_{ȷ}},{𝔗}_{ȷ})],[({𝕁}_{{𝔲}_{ȷ}},{𝔘}_{ȷ}),\left({𝕁}_{y_{ȷ}},Y_{ȷ})\right])\left(ȷ=1,2,\ldots ,\beta \right)$ be a set of 2TLIV*q*-ROFNs, and let $R^{-}=([\underset{ȷ}{\min}({𝕁}_{{𝔯}_{ȷ}},{ℜ}_{ȷ}),\min_{ȷ}\left({𝕁}_{{𝔱}_{ȷ}},{𝔗}_{ȷ}\right)],[\underset{ȷ}{\max}\left({𝕁}_{{𝔲}_{ȷ}},{𝔘}_{ȷ}\right)$, $\underset{ȷ}{\max}\left({𝕁}_{y_{ȷ}},Y_{ȷ})]\right)$ and $R^{+}=(\left[\underset{ȷ}{\max}\left({𝕁}_{{𝔯}_{ȷ}},{ℜ}_{ȷ}\right),\underset{ȷ}{\max}\left({𝕁}_{{𝔱}_{ȷ}},{𝔗}_{ȷ}\right)\right],$ $\left[\underset{ȷ}{\min}\left({𝕁}_{{𝔲}_{ȷ}},{𝔘}_{ȷ}\right),\underset{ȷ}{\min}\left({𝕁}_{y_{ȷ}},Y_{ȷ}\right)\right]$), then
$$\begin{array}{l} R^{-}\leq \text{2TLIV}q\text{-ROFWA}\left(R_{1},R_{2},\ldots ,R_{\beta }\right)\leq R^{+}. \end{array}$$

Definition 10. Consider $R_{ȷ}=\left(\left[\left({𝕁}_{{𝔯}_{ȷ}},{ℜ}_{ȷ}\right),\left({𝕁}_{{𝔱}_{ȷ}},{𝔗}_{ȷ}\right)\right],\left[\left({𝕁}_{{𝔲}_{ȷ}},{𝔘}_{ȷ}\right),\left({𝕁}_{y_{ȷ}},Y_{ȷ}\right)\right]\right)\left(ȷ=1,2,\ldots ,\beta \right)$ be a set of 2TLIV*q*-ROFNs. The 2TLIV*q*-ROFWJ operator represents a transformation that ensures ⊺^β^ → ⊺ is transformed as follows in Equation 8:


(8) $$\begin{array}{l} \text{2TL}T\text{-SFWJ}\left(R_{1},R_{2},\ldots ,R_{\beta }\right)={⊗}_{ȷ=1}^{\beta }R_{ȷ}^{\varpi_{ȷ}}, \end{array}$$

in which ⊺ is the set of 2TLIV*q*-ROFNs, $\varpi ={\left(\varpi_{1},\varpi_{2},\ldots ,\varpi_{\beta }\right)}^{T}$ is the weighting vector of $R_{ȷ}\left(ȷ=1,2,\ldots ,\beta \right)$, such that ϖ_ȷ_ ∈ [0, 1] and $\overset{\beta }{\underset{ȷ=1}{\sum }}\varpi_{ȷ}=1.$

Theorem 3. Consider $R_{ȷ}=\left(\left[\left({𝕁}_{{𝔯}_{ȷ}},{ℜ}_{ȷ}\right),\left({𝕁}_{{𝔱}_{ȷ}},{𝔗}_{ȷ}\right)\right],\left[\left({𝕁}_{{𝔲}_{ȷ}},{𝔘}_{ȷ}\right),\left({𝕁}_{y_{ȷ}},Y_{ȷ}\right)\right]\right)\left(ȷ=1,2,\ldots ,\beta \right)$ be a group of 2TLIV*q*-ROFNs regarding a weight vector $\varpi ={\left(\varpi_{1},\varpi_{2},\ldots ,\varpi_{\beta }\right)}^{T}$, such that ϖ_ȷ_ ∈ [0, 1] and $\overset{\beta }{\underset{ȷ=1}{\sum }}\varpi_{ȷ}=1$. The aggregate value obtained through the 2TLIV*q*-ROFWJ operator remains a 2TLIVq-ROFN, and the 2TLIVq-ROFWJ operator represents in the Equation 9.


(9) $$\begin{array}{l} \text{2TLIV}q\text{-ROFWJ}\left(R_{1},R_{2},\ldots ,R_{\beta }\right)\\ =\left(\begin{array}{l} \left[\begin{array}{l} \Delta \left(\tau \overset{\beta }{\underset{ȷ=1}{\prod }}{\left(\frac{\Delta^{-1}\left({𝕁}_{{𝔯}_{ȷ}},{ℜ}_{ȷ}\right)}{\tau }\right)}^{\varpi_{ȷ}}\right),\Delta \left(\tau \overset{\beta }{\underset{ȷ=1}{\prod }}{\left(\frac{\Delta^{-1}\left({𝕁}_{{𝔱}_{ȷ}},{𝔗}_{ȷ}\right)}{\tau }\right)}^{\varpi_{ȷ}}\right) \end{array}\right],\\ \left[\begin{array}{l} \Delta \left(\tau \left(\sqrt[{q}]{1-\overset{\beta }{\underset{ȷ=1}{\prod }}{\left(1-{\left(\frac{\Delta^{-1}\left({𝕁}_{{𝔲}_{ȷ}},{𝔘}_{ȷ}\right)}{\tau }\right)}^{q}\right)}^{\varpi_{ȷ}}}\right)\right),\Delta \left(\tau \left(\sqrt[{q}]{1-\overset{\beta }{\underset{ȷ=1}{\prod }}{\left(1-{\left(\frac{\Delta^{-1}\left({𝕁}_{y_{ȷ}},Y_{ȷ}\right)}{\tau }\right)}^{q}\right)}^{\varpi_{ȷ}}}\right)\right) \end{array}\right] \end{array}\right). \end{array}$$

The demonstration closely resembles the one found in Theorems 1 and 2.

## 5 Methodology

Research methodology in decision-making is the systematic process of gathering and analyzing information to inform a choice. It involves defining the problem, choosing a research design to collect data, analyzing that data, and using the results to weigh options and make a well-supported decision. An essential subfield of MADM research, MAGDM entails a group of DMs evaluating alternatives based on multiple competing attributes for ranking all the alternatives or choosing the most suitable one. Recent articles on MAGDM have emphasized methodological techniques for ambiguous situations in general (Ozcalici, 2022; Ashouri et al., 2023; Colombo et al., 2023; Kiptum et al., 2023; Lazarashouri and Najafi, 2024). Due to the severity of MAGDM challenges, it is difficult for DMs to collect all possible alternative information. Understanding how to deal with uncertainty and ambiguity is crucial for selecting the best option in practical decision-making challenges. Innovative MABAC method facilitates group decision-making by offering a structured framework that integrates multiple attributes, fostering collaboration and ensuring comprehensive evaluation of alternatives. MABAC helps consider uncertainties in preferences by incorporating fuzzy logic concepts, ultimately aiming to identify the best alternative through a structured comparison process. In order to assess the linguistic information, a novel 2TLIV*q*-ROF-MABAC method is constructed in which the collected data is aggregated by the 2TLIV*q*-ROFWA and 2TLIV*q*-ROFWJ operators. In particular, to cope with MAGDM problems, there is a set of α alternatives Γ = {Γ_1_, Γ_2_, …, Γ_α_}, β attributes Θ = {Θ_1_, Θ_2_, …, Θ_β_}, and 𝔣 experts $E=\left\{E_{1},E_{2},\ldots ,E_{𝔣}\right\}$, and let $\varpi ={\left(\varpi_{1},\varpi_{2},\ldots ,\varpi_{\beta }\right)}^{T}$ and $\omega^{\prime }={\left(\omega_{1}^{\prime },\omega_{2}^{\prime },\ldots ,\omega_{𝔣}^{\prime }\right)}^{T}$ be the weight vector of the Θ_ȷ_ and weight vector of the $E_{\gamma }$ satisfy ϖ_ȷ_ ∈ [0, 1], ω_γ_ ∈ [0, 1], $\sum_{ȷ=1}^{\beta }\varpi_{ȷ}=1$, and $\sum_{\gamma =1}^{𝔣}\omega_{\gamma }=1$.

**Step 1**. For 2TLIV*q*-ROFS, the evaluation matrix is constructed as $R={\left[{ℶ}_{ıȷ}^{\gamma }\right]}_{\alpha \times \beta }=$$\left({\left[\left(𝕁{𝔯}_{ıȷ},{ℜ}_{ıȷ}\right),\left({𝕁}_{{𝔱}_{ıȷ}},{𝔗}_{ıȷ}\right)\right]}^{\gamma },{\left[\left({𝕁}_{{𝔲}_{ıȷ}},{𝔘}_{ıȷ}\right),\left({𝕁}_{y_{ıȷ}},Y_{ıȷ}\right)\right]}^{\gamma }\right)$(ı=1,2,…,α,ȷ=1,2,…,β,γ=1,2,…,𝔣). The 2TLIV*q*-ROFS conveys the information regarding alternatives ${ℶ}_{ı}$ on attributes Θ_ȷ_ as evaluated by DMs $E_{\gamma }$.
**Step 2**. By utilizing the 2TLIV*q*-ROFWA operator from Equation (8), the combined 2TLIV*q*-ROFNs matrix *r* = [${ℶ}_{ı}$_*ȷ*_]_α×β_ is obatined, in which ${ℶ}_{ıȷ}=\left(\left[\left({𝕁}_{{𝔯}_{ıȷ}},{ℜ}_{ıȷ}\right),\left({𝕁}_{{𝔱}_{ıȷ}},{𝔗}_{ıȷ}\right)\right],\left[\left({𝕁}_{{𝔲}_{ıȷ}},{𝔘}_{ıȷ}\right),\left({𝕁}_{y_{ıȷ}},Y_{ıȷ}\right)\right]\right)$.
**Step 3**. Applying an attribute-based procedure, normalize the aggregated matrix *r* = [${ℶ}_{ı}$_*ȷ*_]_α×β_ by using Equations 10 and 11.
  For benefit attributes:
(10) $$\begin{array}{c} {\mathbb{N}}_{ıȷ}={ℶ}_{ıȷ}=([({𝕁}_{r_{ıȷ}},{ℜ}_{ıȷ}),({𝕁}_{t_{ıȷ}},T_{ıȷ})],[({𝕁}_{u_{ıȷ}},\\ U_{ıȷ}),({𝕁}_{y_{ıȷ}},Y_{ıȷ})]),\;ıȷ=1,2,\ldots ,\alpha ,\;=1,2,\ldots ,\beta \end{array}$$
  For cost attributes:
(11) $$\begin{array}{c} {\mathbb{N}}_{ıȷ}={ℶ}_{ıȷ}^{c}=[({𝕁}_{u_{ıȷ}},U_{ıȷ}),({𝕁}_{y_{ıȷ}},Y_{ıȷ})],[({𝕁}_{r_{ıȷ}},{ℜ}_{ıȷ}),({𝕁}_{t_{ıȷ}},\\ T_{ıȷ})],ıȷ=1,2,\ldots ,\alpha ,=1,2,\ldots ,\beta \end{array}$$
**Step 4**. By using the obtained results of normalized matrix ${\mathbb{N}}_{ıȷ}=\left(\left[\left({𝕁}_{{𝔯}_{ıȷ}},{ℜ}_{ıȷ}\right),\left({𝕁}_{{𝔱}_{ıȷ}},{𝔗}_{ıȷ}\right)\right],\left[\left({𝕁}_{{𝔲}_{ıȷ}},{𝔘}_{ıȷ}\right),\left({𝕁}_{y_{ıȷ}},Y_{ıȷ}\right)\right]\right)$ and attribute weights ϖ_ȷ_(ȷ = 1, 2, …, β), the 2TLIV*q*-ROF weighted normalized matrix ${𝕎\mathbb{N}}_{ıȷ}=\left(\left[\left({𝕁}_{{𝔯}_{ıȷ}}^{\prime },{ℜ}_{ıȷ}^{\prime }\right),\left({𝕁}_{{𝔱}_{ıȷ}}^{\prime },{𝔗}_{ıȷ}^{\prime }\right)\right],\left[\left({𝕁}_{{𝔲}_{ıȷ}}^{\prime },{𝔘}_{ıȷ}^{\prime }\right),\left({𝕁}_{y_{ıȷ}}^{\prime },Y_{ıȷ}^{\prime }\right)\right]\right)$ is put forward as in Equation 12:
(12) $$\begin{array}{l} {𝕎\mathbb{N}}_{ıȷ}=\varpi_{ȷ}⊗{\mathbb{N}}_{ıȷ}==\left(\begin{array}{l} \left[\begin{array}{l} \Delta \left(\tau \left(\sqrt[{q}]{1-{\left(1-{\left(\frac{\Delta^{-1}\left({𝕁}_{𝔯},ℜ\right)}{\tau }\right)}^{q}\right)}^{\varpi_{ȷ}}}\right)\right),\Delta \left(\tau \left(\sqrt[{q}]{1-{\left(1-{\left(\frac{\Delta^{-1}\left({𝕁}_{𝔱},𝔗\right)}{\tau }\right)}^{q}\right)}^{\varpi_{ȷ}}}\right)\right) \end{array}\right],\\ \left[\begin{array}{l} \Delta \left(\tau \left({\left(\frac{\Delta^{-1}\left({𝕁}_{𝔲},𝔘\right)}{\tau }\right)}^{\varpi_{ȷ}}\right)\right),\Delta \left(\tau \left({\left(\frac{\Delta^{-1}\left({𝕁}_{y},Y\right)}{\tau }\right)}^{\varpi_{ȷ}}\right)\right) \end{array}\right] \end{array}\right). \end{array}$$
**Step 5**. Calculate BAA values as well as matrix ***G*** = [***g***_ȷ_]_1×β_ is constructed into the following form by using Equation 13;
(13) $$\begin{array}{l} g_{ȷ}={\left(\overset{\alpha }{\underset{ı=1}{\prod }}{𝕎\mathbb{N}}_{ıȷ}\right)}^{1/\alpha }=\left(\begin{array}{l} \left[\begin{array}{l} \Delta \left(\tau \overset{\beta }{\underset{ȷ=1}{\prod }}{\left(\frac{\Delta^{-1}\left({𝕁}_{{𝔯}_{ȷ}}^{\prime },{ℜ}_{ȷ}^{\prime }\right)}{\tau }\right)}^{1/\alpha }\right),\Delta \left(\tau \overset{\beta }{\underset{ȷ=1}{\prod }}{\left(\frac{\Delta^{-1}\left({𝕁}_{{𝔱}_{ȷ}}^{\prime },{𝔗}_{ȷ}^{\prime }\right)}{\tau }\right)}^{1/\alpha }\right) \end{array}\right],\\ \left[\begin{array}{l} \Delta \left(\tau \left(\sqrt[{q}]{1-\overset{\beta }{\underset{ȷ=1}{\prod }}{\left(1-{\left(\frac{\Delta^{-1}\left({𝕁}_{{𝔲}_{ȷ}}^{\prime },{𝔘}_{ȷ}^{\prime }\right)}{\tau }\right)}^{q}\right)}^{1/\alpha }}\right)\right),\\ \Delta \left(\tau \left(\sqrt[{q}]{1-\overset{\beta }{\underset{ȷ=1}{\prod }}{\left(1-{\left(\frac{\Delta^{-1}\left({𝕁}_{y_{ȷ}}^{\prime },Y_{ȷ}^{\prime }\right)}{\tau }\right)}^{q}\right)}^{1/\alpha }}\right)\right) \end{array}\right] \end{array}\right). \end{array}$$
**Step 6**. The distance matrix $D={\left[d_{ıȷ}\right]}_{\alpha \times \beta }$ is constructed from the results of ${𝕎\mathbb{N}}_{ı}$_*ȷ*_ and ***G***_ȷ_ matrices by calculating the 2TLIV*q*-ROF normalized Hamming distance described in Equation (5).
(14) $$\begin{array}{l} d_{ıȷ}=\{\begin{array}{l} d\left({𝕎\mathbb{N}}_{ıȷ},g_{ȷ}\right),\;if\;{𝕎\mathbb{N}}_{ıȷ}>g_{ȷ}\\ \;0,\;if\;{𝕎\mathbb{N}}_{ıȷ}=g_{ȷ}\\ -d\left({𝕎\mathbb{N}}_{ıȷ},g_{ȷ}\right),\;if\;{𝕎\mathbb{N}}_{ıȷ}<g_{ȷ} \end{array} \end{array}$$
**Step 7**. The cumulative values of the $d_{ı}$_*ȷ*_ for each alternative can be calculated by using Equation 15.
(15) $$\begin{array}{l} S_{ı}=\overset{\beta }{\underset{ȷ=1}{\sum }}d_{ıȷ} \end{array}$$
  The comprehensive assessment result $S_{ı}$ can be used to establish the order of all alternatives; obviously, the better the decision, the greater the comprehensive assessment result $S_{ı}$.

**Figure 1:**
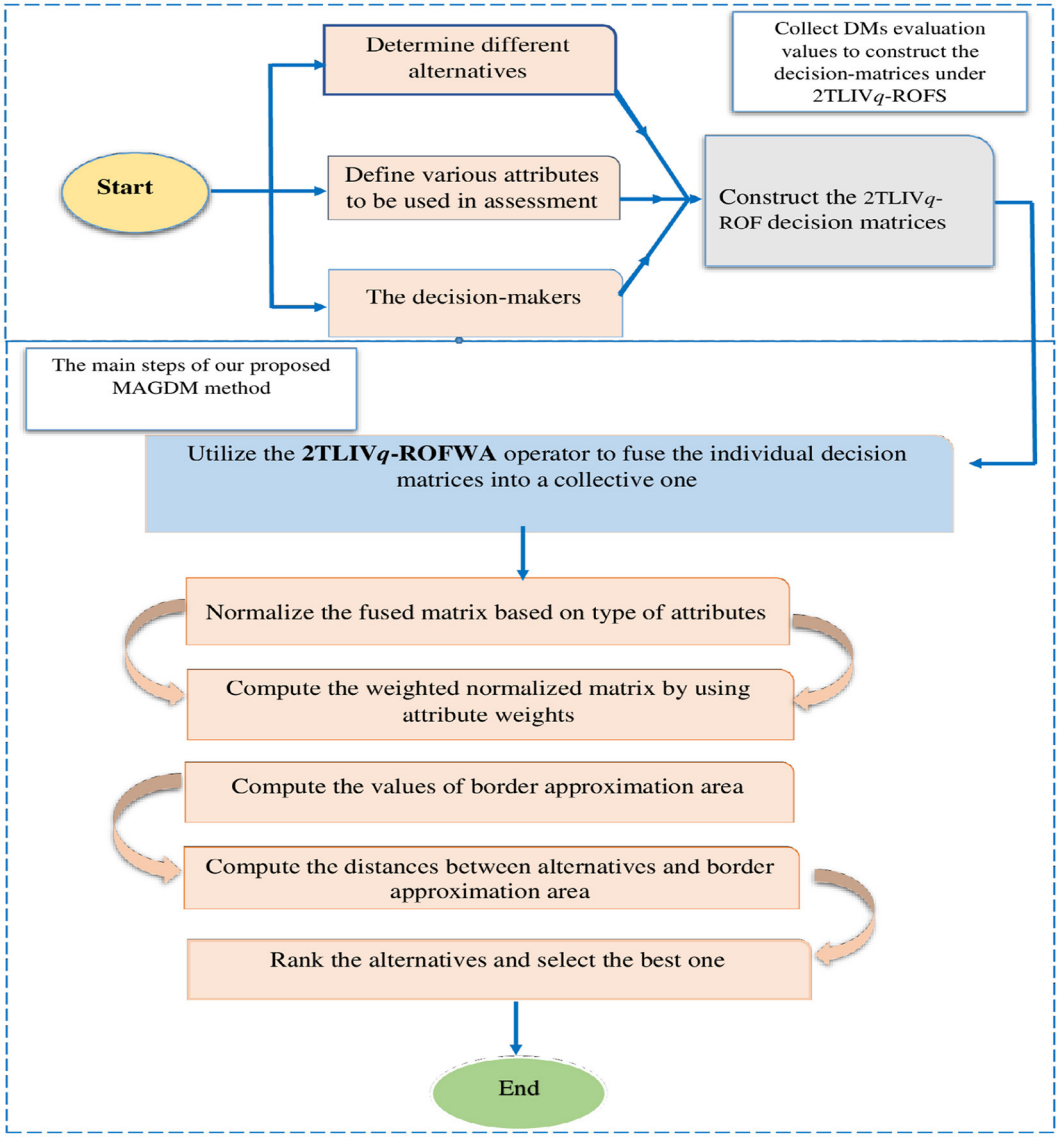
A graphical representation illustrating the methodology.

Figure 1 provides a visual representation of the methodology.

## 6 Case study

A case study follows to demonstrate the usefulness and versatility of the proposed approach. We validate our strategy through the challenging process of choosing the best breast cancer treatment.

### 6.1 The problem description

Breast cancer is when cells in the breast tissue turn harmful and damage the tissue. Although it is more prevalent among women, men are not impervious to this particular form of cancer. A multidisciplinary treatment approach is required due to the complexity of breast cancer. Numerous factors play a role in the decision of which treatment is best for each patient. These include the stage and subtype of the cancer, the patient's age and overall health, the patient's personal preferences and values, as well as the availability and accessibility of diverse treatment options. Antineoplastic agents, targeted therapies, radiation therapy, chemotherapy, hormone therapy, and immunotherapy are among the most frequently used treatments for breast cancer. Each of these treatments possesses its own set of benefits and drawbacks and may be implemented individually or in combination with other therapies. The objectives of treatment are to eliminate or manage the cancer, safeguard against its recurrence or growth, and improve the overall wellbeing and survival of the patient. The formulation of a treatment plan for a patient with breast cancer is a complex and individualized process that considers numerous factors and potentialities. Different types of cancer treatment options are considered based on the cancer's type, stage, and characteristics, as well as the patient's preferences and overall health. Some people have access to the following possibilities for deciding how to treat their breast cancer:

1. **Lumpectomy (Γ_1_)**
  In this procedure, the majority of the breast is left untouched, and just the tumor and a narrow margin of healthy tissue surrounding it are removed. Radiation therapy is frequently used after a lumpectomy to eradicate any breast cancer cells that may still be present. If a patient has a tiny or early-stage tumor and wants to maintain the appearance and function of their breasts, a lumpectomy may be a possibility.
2. **Mastectomy (Γ_2_)**
  In this procedure, the entire breast-along with the nipple and areola is removed. Patients with big, aggressive, or multifocal tumors, those who have a high chance of recurrence, or those who have a genetic mutation that raises their risk of getting breast cancer may be advised to have a mastectomy. A mastectomy may be performed in conjunction with reconstructive surgery to reshape the breasts using implants or body tissue.
3. **Chemotherapy (Γ_3_)**
  The goal of this therapy is to eradicate cancer cells throughout the body via medication. Prior to surgery (neoadjuvant) to reduce the size of the tumor and make it simpler to remove, or after surgery (adjuvant) to lower the risk of growth or recurrence, chemotherapy may be administered. Chemotherapy can relieve symptoms and improve the quality of life in advanced or cancerous breast cancer patients.
4. **Hormone therapy (Γ_4_)**
  This therapy inhibits or reduces the levels of the hormones (estrogen and progesterone) that encourage the growth of some kinds of breast cancer cells. Whether used alone or in conjunction with other therapies, hormone therapy can be administered as tablets, injections, or implants. Patients identified as hormone receptor-positive breast tumors, constituting an estimated 70% of the total breast cancer cases, may qualify for hormone therapy.
5. **Targeted therapy (Γ_5_)**
  Drugs that specifically target substances or processes connected with the advancement and survival of cancer cells are used in this therapy. Since chemotherapy affects normal cells less, targeted treatment could have fewer negative effects than chemotherapy. Patients with particular forms of breast cancer, such as HER2-positive breast cancer, that have particular genetic alterations or indicators, may have the option of targeted therapy.
6. **Immunotherapy (Γ_6_)**
  The immune system is encouraged by the use of medicines in this therapy to identify and fight cancer cells. Immunotherapy may strengthen the body's natural defenses, and inhibit signals that enable cancer cells to avoid detection, or transfer poisons or radioactive materials directly to cancer cells. For individuals with certain kinds of breast cancer, such as triple-negative breast cancer, that has unique traits or mutations, immunotherapy may be a possibility.
7. **Clinical trials (Γ_7_)**
  These trials examine the safety and efficacy of novel medications, apparatus, methods, or treatment accumulation in humans. Access to novel or experimental medicines that are still only available in research settings may be provided via clinical trials. Patients who have tried all of the traditional therapies, have uncommon or difficult-to-treat kinds of breast cancer, or who want to help progress science and medicine may be candidates for clinical trials.

The selection of breast cancer treatments is an intricate procedure involving multiple factors and stakeholders. Consequently, breast cancer treatment selection can be regarded as a conventional MAGDM problem. We intend to evaluate breast cancer treatment utilizing the 2TLIV*q*-ROF-MABAC method that is suggested in this paper and data is aggregated by the proposed 2TLIV*q*-ROF aggregation operators. In this regard, seven distinct alternatives can be considered (a brief discussion of each is given above) Γ = {Γ_1_, Γ_2_, Γ_3_, …, Γ_7_}, evaluated by an advisory group of four DMs $E=\left\{E_{1},E_{2},E_{3},E_{4}\right\}$ with weights ω′ = (0.2192, 0.2134, 0.1930, 0.1906)^*T*^ with the goal to deal with the problem described above. The four DMs choose an optimal alternative based on the four attributes Θ = {Θ_1_, Θ_2_, Θ_3_, Θ_4_} (as depicted in Table 3) and their corresponding weights are, ϖ = (0.2542, 0.2533, 0.2480, 0.2445)^*T*^. To evaluate the significance of each LTS 𝔏, four DMs express their assessments. The LTS categories include 𝔏 = {𝕁_0_: Immaterial, 𝕁_1_: Low Suitability, 𝕁_2_: Moderate Suitability, 𝕁_3_: High Suitability, 𝕁_4_: Reasonable, 𝕁_5_: Low Efficacy, 𝕁_6_: Moderate Efficacy, 𝕁_7_: High Efficacy, 𝕁_8_: Trustable }.

**Table 3:** A synopsis of assessing attributes.

**Attributes**	**Description**
The stage and subtype of cancer (Θ_1_)	This determines the extent of the illness, its prognosis, and the best course of therapy. Different breast cancer subtypes exhibit diverse genomic traits, biological activities, and therapeutic responses.
The patient's preferences and goals (Θ_2_)	This includes discussing the benefits and drawbacks of various forms of treatment, including surgical procedures, chemotherapy, radiation therapy, hormone therapy, and targeted therapy, among other types of treatment. It is possible that the patient's age, health, quality of life, fertility, and personal values will all impact the decisions they make and their goals they set for themselves.
The availability and accessibility of resources (Θ_3_)	This refers to the availability and accessibility of trained healthcare professionals, facilities, tools, and other support services that are required to offer high-quality, evidence-based treatment. Depending on the patient's geographic location, socioeconomic level, insurance status, and transportation alternatives, the services that are available and accessible may differ.
The potential short-term and long-term outcomes (Θ_4_)	This involves assessing the predicted efficacy and toxicity of various treatment approaches as well as their effects on survival, recurrence, morbidity, and psychological wellbeing. The future results may rely on the tumor features, treatment response, side effects, comorbidities, and adherence to follow-up care.

Tables 4–7 provide decision matrices determined by each of the four DMs' assessment values for all alternatives. In these tables, the 2TL term (𝕁_ℓ_, 0) can be written as 𝕁_ℓ_ for convenience.

**Table 4:** 2TLIV*q*-ROF decision matrix by $E_{1}$.

	**Θ_1_**	**Θ_2_**	**Θ_3_**	**Θ_4_**
Γ_1_	([𝕁_2_, 𝕁_3_], [𝕁_1_, 𝕁_4_])	([𝕁_3_, 𝕁_4_], [𝕁_2_, 𝕁_5_])	([𝕁_1_, 𝕁_3_], [𝕁_5_, 𝕁_6_])	([𝕁_3_, 𝕁_4_], [𝕁_1_, 𝕁_2_])
Γ_2_	([𝕁_3_, 𝕁_5_], [𝕁_4_, 𝕁_5_])	([𝕁_4_, 𝕁_5_], [𝕁_3_, 𝕁_4_])	([𝕁_1_, 𝕁_7_], [𝕁_2_, 𝕁_4_])	([𝕁_4_, 𝕁_5_], [𝕁_3_, 𝕁_5_])
Γ_3_	([𝕁_5_, 𝕁_7_], [𝕁_3_, 𝕁_4_])	([𝕁_2_, 𝕁_5_], [𝕁_3_, 𝕁_6_])	([𝕁_4_, 𝕁_5_], [𝕁_3_, 𝕁_4_])	([𝕁_4_, 𝕁_5_], [𝕁_1_, 𝕁_7_])
Γ_4_	([𝕁_2_, 𝕁_4_], [𝕁_3_, 𝕁_6_])	([𝕁_1_, 𝕁_6_], [𝕁_5_, 𝕁_6_])	([𝕁_4_, 𝕁_6_], [𝕁_5_, 𝕁_7_])	([𝕁_2_, 𝕁_4_], [𝕁_1_, 𝕁_2_])
Γ_5_	([𝕁_3_, 𝕁_4_], [𝕁_2_, 𝕁_6_])	([𝕁_4_, 𝕁_5_], [𝕁_3_, 𝕁_4_])	([𝕁_2_, 𝕁_3_], [𝕁_4_, 𝕁_7_])	([𝕁_2_, 𝕁_4_], [𝕁_1_, 𝕁_3_])
Γ_6_	([𝕁_1_, 𝕁_3_], [𝕁_2_, 𝕁_5_])	([𝕁_1_, 𝕁_4_], [𝕁_2_, 𝕁_3_])	([𝕁_1_, 𝕁_3_], [𝕁_6_, 𝕁_7_])	([𝕁_3_, 𝕁_5_], [𝕁_1_, 𝕁_3_])
Γ_7_	([𝕁_3_, 𝕁_4_], [𝕁_5_, 𝕁_6_])	([𝕁_3_, 𝕁_5_], [𝕁_4_, 𝕁_5_])	([𝕁_3_, 𝕁_4_], [𝕁_2_, 𝕁_4_])	([𝕁_4_, 𝕁_5_], [𝕁_4_, 𝕁_5_])

**Table 5:** 2TLIV*q*-ROF decision matrix by $E_{2}$.

	**Θ_1_**	**Θ_2_**	**Θ_3_**	**Θ_4_**
Γ_1_	([𝕁_3_, 𝕁_4_], [𝕁_2_, 𝕁_3_])	([𝕁_2_, 𝕁_3_], [𝕁_4_, 𝕁_5_])	([𝕁_2_, 𝕁_3_], [𝕁_4_, 𝕁_5_])	([𝕁_3_, 𝕁_4_], [𝕁_3_, 𝕁_6_])
Γ_2_	([𝕁_2_, 𝕁_3_], [𝕁_3_, 𝕁_4_])	([𝕁_2_, 𝕁_4_], [𝕁_1_, 𝕁_3_])	([𝕁_1_, 𝕁_4_], [𝕁_2_, 𝕁_5_])	([𝕁_3_, 𝕁_4_], [𝕁_3_, 𝕁_4_])
Γ_3_	([𝕁_2_, 𝕁_4_], [𝕁_3_, 𝕁_5_])	([𝕁_2_, 𝕁_4_], [𝕁_5_, 𝕁_6_])	([𝕁_3_, 𝕁_4_], [𝕁_4_, 𝕁_5_])	([𝕁_2_, 𝕁_3_], [𝕁_2_, 𝕁_4_])
Γ_4_	([𝕁_4_, 𝕁_5_], [𝕁_1_, 𝕁_2_])	([𝕁_3_, 𝕁_4_], [𝕁_4_, 𝕁_5_])	([𝕁_3_, 𝕁_4_], [𝕁_2_, 𝕁_4_])	([𝕁_2_, 𝕁_4_], [𝕁_1_, 𝕁_3_])
Γ_5_	([𝕁_2_, 𝕁_3_], [𝕁_4_, 𝕁_5_])	([𝕁_1_, 𝕁_4_], [𝕁_2_, 𝕁_3_])	([𝕁_2_, 𝕁_3_], [𝕁_1_, 𝕁_2_])	([𝕁_3_, 𝕁_4_], [𝕁_1_, 𝕁_5_])
Γ_6_	([𝕁_3_, 𝕁_4_], [𝕁_2_, 𝕁_4_])	([𝕁_1_, 𝕁_3_], [𝕁_2_, 𝕁_3_])	([𝕁_4_, 𝕁_5_], [𝕁_1_, 𝕁_2_])	([𝕁_1_, 𝕁_2_], [𝕁_3_, 𝕁_4_])
Γ_7_	([𝕁_4_, 𝕁_7_], [𝕁_5_, 𝕁_7_])	([𝕁_1_, 𝕁_3_], [𝕁_4_, 𝕁_6_])	([𝕁_1_, 𝕁_3_], [𝕁_2_, 𝕁_3_])	([𝕁_2_, 𝕁_4_], [𝕁_2_, 𝕁_4_])

**Table 6:** 2TLIV*q*-ROF decision matrix by $E_{3}$.

	**Θ_1_**	**Θ_2_**	**Θ_3_**	**Θ_4_**
Γ_1_	([𝕁_2_, 𝕁_3_], [𝕁_3_, 𝕁_4_])	([𝕁_3_, 𝕁_4_], [𝕁_4_, 𝕁_5_])	([𝕁_1_, 𝕁_2_], [𝕁_3_, 𝕁_4_])	([𝕁_2_, 𝕁_4_], [𝕁_2_, 𝕁_3_])
Γ_2_	([𝕁_1_, 𝕁_4_], [𝕁_2_, 𝕁_3_])	([𝕁_2_, 𝕁_5_], [𝕁_1_, 𝕁_2_])	([𝕁_2_, 𝕁_3_], [𝕁_3_, 𝕁_4_])	([𝕁_4_, 𝕁_5_], [𝕁_4_, 𝕁_6_])
Γ_3_	([𝕁_3_, 𝕁_5_], [𝕁_1_, 𝕁_2_])	([𝕁_1_, 𝕁_3_], [𝕁_3_, 𝕁_5_])	([𝕁_3_, 𝕁_4_], [𝕁_2_, 𝕁_5_])	([𝕁_4_, 𝕁_5_], [𝕁_2_, 𝕁_5_])
Γ_4_	([𝕁_1_, 𝕁_5_], [𝕁_2_, 𝕁_4_])	([𝕁_3_, 𝕁_4_], [𝕁_2_, 𝕁_4_])	([𝕁_2_, 𝕁_3_], [𝕁_1_, 𝕁_4_])	([𝕁_3_, 𝕁_4_], [𝕁_5_, 𝕁_7_])
Γ_5_	([𝕁_2_, 𝕁_3_], [𝕁_4_, 𝕁_5_])	([𝕁_1_, 𝕁_3_], [𝕁_1_, 𝕁_4_])	([𝕁_1_, 𝕁_2_], [𝕁_4_, 𝕁_5_])	([𝕁_5_, 𝕁_6_], [𝕁_2_, 𝕁_3_])
Γ_6_	([𝕁_3_, 𝕁_4_], [𝕁_2_, 𝕁_3_])	([𝕁_1_, 𝕁_3_], [𝕁_2_, 𝕁_3_])	([𝕁_4_, 𝕁_5_], [𝕁_1_, 𝕁_2_])	([𝕁_2_, 𝕁_3_], [𝕁_3_, 𝕁_4_])
Γ_7_	([𝕁_2_, 𝕁_3_], [𝕁_2_, 𝕁_4_])	([𝕁_1_, 𝕁_3_], [𝕁_2_, 𝕁_3_])	([𝕁_5_, 𝕁_6_], [𝕁_3_, 𝕁_4_])	([𝕁_3_, 𝕁_4_], [𝕁_2_, 𝕁_3_])

**Table 7:** 2TLIV*q*-ROF decision matrix by $E_{4}$.

	**Θ_1_**	**Θ_2_**	**Θ_3_**	**Θ_4_**
Γ_1_	([𝕁_2_, 𝕁_3_], [𝕁_3_, 𝕁_5_])	([𝕁_3_, 𝕁_4_], [𝕁_1_, 𝕁_2_])	([𝕁_2_, 𝕁_3_], [𝕁_1_, 𝕁_2_])	([𝕁_1_, 𝕁_4_], [𝕁_3_, 𝕁_5_])
Γ_2_	([𝕁_1_, 𝕁_2_], [𝕁_2_, 𝕁_4_])	([𝕁_2_, 𝕁_3_], [𝕁_4_, 𝕁_5_])	([𝕁_4_, 𝕁_5_], [𝕁_3_, 𝕁_6_])	([𝕁_3_, 𝕁_4_], [𝕁_2_, 𝕁_4_])
Γ_3_	([𝕁_4_, 𝕁_5_], [𝕁_3_, 𝕁_4_])	([𝕁_2_, 𝕁_3_], [𝕁_1_, 𝕁_2_])	([𝕁_1_, 𝕁_4_], [𝕁_2_, 𝕁_3_])	([𝕁_2_, 𝕁_4_], [𝕁_3_, 𝕁_5_])
Γ_4_	([𝕁_3_, 𝕁_4_], [𝕁_3_, 𝕁_5_])	([𝕁_2_, 𝕁_4_], [𝕁_3_, 𝕁_4_])	([𝕁_2_, 𝕁_4_], [𝕁_1_, 𝕁_2_])	([𝕁_1_, 𝕁_2_], [𝕁_1_, 𝕁_3_])
Γ_5_	([𝕁_2_, 𝕁_3_], [𝕁_4_, 𝕁_5_])	([𝕁_2_, 𝕁_6_], [𝕁_1_, 𝕁_2_])	([𝕁_3_, 𝕁_4_], [𝕁_4_, 𝕁_6_])	([𝕁_5_, 𝕁_7_], [𝕁_6_, 𝕁_7_])
Γ_6_	([𝕁_3_, 𝕁_4_], [𝕁_4_, 𝕁_5_])	([𝕁_2_, 𝕁_3_], [𝕁_1_, 𝕁_2_])	([𝕁_5_, 𝕁_6_], [𝕁_1_, 𝕁_2_])	([𝕁_1_, 𝕁_2_], [𝕁_2_, 𝕁_4_])
Γ_7_	([𝕁_2_, 𝕁_3_], [𝕁_4_, 𝕁_5_])	([𝕁_3_, 𝕁_4_], [𝕁_2_, 𝕁_4_])	([𝕁_4_, 𝕁_5_], [𝕁_4_, 𝕁_7_])	([𝕁_2_, 𝕁_3_], [𝕁_2_, 𝕁_4_])

### 6.2 Evaluation of case study utilizing the proposed 2TLIV*q*-ROF approach

The 2TLIV*q*-ROF-MABAC approach based on the 2TLIV*q*-ROFWA operator is used in this subsection to illustrate the assessment procedure for selecting the best cancer treatment.

**Step 1**. We construct the 2TLIV*q*-ROF assessment matrices $R^{\kappa }={\left[{ℶ}_{ıȷ}^{\kappa }\right]}_{7\times 4}=\left({\left[\left({𝕁}_{{𝔯}_{ıȷ}},{ℜ}_{ıȷ}\right),\left({𝕁}_{{𝔱}_{ıȷ}},{𝔗}_{ıȷ}\right)\right]}^{\gamma },{\left[\left({𝕁}_{{𝔲}_{ıȷ}},{𝔘}_{ıȷ}\right),\left({𝕁}_{y_{ıȷ}},Y_{ıȷ}\right)\right]}^{\gamma }\right)$ (ı = 1, 2, …, 7, ȷ = 1, 2, 3, 4, γ = 1, 2, 3, 4) as shown in Tables 4–7.
**Step 2**. The combined 2TLIV*q*-ROFNs matrix *r* = [${ℶ}_{ı}$_*ȷ*_]_7×4_ is obtained utilizing the 2TLIV*q*-ROFWA aggregation operator, which is illustrated in Table 8 [assuming that *q* = 6, τ = 8, ω′ = (0.2192, 0.2134, 0.1930, 0.1906)^*T*^].
**Step 3**. Normalization is unnecessary provided all of the attributes are of the benefit type. Consider the evaluation matrix presented in Table 8 to be the normalized version.
**Step 4**. In accordance with ${\mathbb{N}}_{ı}$_*ȷ*_ = ([($=(\left[\left({𝕁}_{{𝔯}_{ıȷ}},{ℜ}_{ıȷ}\right),\left({𝕁}_{{𝔱}_{ıȷ}},{𝔗}_{ıȷ}\right)\right]$, $\left[\left({𝕁}_{{𝔲}_{ıȷ}},{𝔘}_{ıȷ}\right),\left({𝕁}_{y_{ıȷ}},Y_{ıȷ}\right)]\right)$ (ı= 1,2,…,7,ȷ= 1,2,3,4) and weights $\varpi_{ȷ}={\left(0.2542,0.2533,0.2480,0.2445\right)}^{T}$, the 2TLIV*q*-ROF weighted normalized matrix ${𝕎\mathbb{N}}_{ıȷ}=\left({\left[\left({𝕁}_{{𝔯}_{ıȷ}}^{\prime },{ℜ}_{ıȷ}^{\prime }\right),\left({𝕁}_{{𝔱}_{ıȷ}}^{\prime },{𝔗}_{ıȷ}^{\prime }\right)\right]}^{\gamma },{\left[\left({𝕁}_{{𝔲}_{ıȷ}}^{\prime },{𝔘}_{ıȷ}^{\prime }\right),\left({𝕁}_{y_{ıȷ}}^{\prime },Y_{ıȷ}^{\prime }\right)\right]}^{\gamma }\right)$(ı = 1, 2, …, 7, ȷ = 1, 2, 3, 4) through Equation (13) can be found in Table 9.
  The scores of 2TLIV*q*-ROF elements of weighted normalized matrix ${𝕎\mathbb{N}}_{ı}$_*ȷ*_ are shown in Table 10.
**Step 5**. Construct the BAA matrix ***G*** = [_***g***_ȷ_]1×4_ using Equation (14). Table 11 displays the results of the BAA matrix ***G***.
**Step 6**. Compute the normalized Hamming distance $D={\left[d_{ıȷ}\right]}_{7\times 4}$ between ${𝕎\mathbb{N}}_{ı}$_*ȷ*_ and ***G***_ȷ_ matrices through the Equation (6) as illustrated in the Table 12.
**Step 7**. The cumulative values of the $d_{ı}$_*ȷ*_ for each alternative are:
$$\begin{array}{l} S_{1}=\overset{4}{\underset{ȷ=1}{\sum }}d_{ıȷ}=0.0234,\;S_{2}=\overset{4}{\underset{ȷ=1}{\sum }}d_{2ȷ}=-0.0184,\\ S_{3}=\overset{4}{\underset{ȷ=1}{\sum }}d_{3ȷ}=-0.0289,\;S_{4}=\overset{4}{\underset{ȷ=1}{\sum }}d_{4ȷ}=0.0569,\\ S_{5}=\overset{4}{\underset{ȷ=1}{\sum }}d_{5ȷ}=0.0043,\;S_{6}=\overset{4}{\underset{ȷ=1}{\sum }}d_{6ȷ}=0.1539,\\ S_{7}=\overset{4}{\underset{ȷ=1}{\sum }}d_{7ȷ}=-0.1199 \end{array}$$

**Table 8:** Combined 2TLIV*q*-ROFN matrix utilizing the 2TLIV*q*-ROFWA operator.

	**Θ_1_**	**Θ_2_**
Γ_1_	([(𝕁_3_, −0.4573), (𝕁_3_, 0.4803), (𝕁_2_, 0.1297), (𝕁_4_, −0.1232)])	([(𝕁_3_, −0.1614), (𝕁_4_, −0.1903), (𝕁_2_, 0.4350), (𝕁_4_, −0.0574)])
Γ_2_	([(𝕁_2_, 0.3407), (𝕁_4_, 0.0405), (𝕁_3_, −0.3995), (𝕁_4_, −0.0911)])	([(𝕁_3_, 0.0869), (𝕁_4_, 0.4974), (𝕁_2_, −0.2203), (𝕁_3_, 0.2979)])
Γ_3_	([(𝕁_4_, 0.0488), (𝕁_6_, −0.2237), (𝕁_2_, 0.3217), (𝕁_4_, −0.3536)])	([(𝕁_2_, −0.0851), (𝕁_4_, 0.1038), (𝕁_3_, −0.3567), (𝕁_4_, 0.3247)])
Γ_4_	([(𝕁_3_, 0.3748), (𝕁_5_, −0.3262), (𝕁_2_, −0.0583), (𝕁_4_, −0.2959)])	([(𝕁_3_, −0.2711), (𝕁_5_, −0.1435), (𝕁_3_, 0.3004), (𝕁_5_, −0.3562)])
Γ_5_	([(𝕁_2_, 0.4091), (𝕁_3_, 0.3437), (𝕁_3_, 0.4882), (𝕁_5_, 0.1833)])	([(𝕁_3_, 0.0663), (𝕁_5_, 0.0882), (𝕁_2_, −0.4600), (𝕁_3_, 0.0569)])
Γ_6_	([(𝕁_3_, −0.1077), (𝕁_4_, −0.1157), (𝕁_2_, 0.3938), (𝕁_4_, 0.1417)])	([(𝕁_2_, −0.3912), (𝕁_3_, 0.3437), (𝕁_2_, −0.3290), (𝕁_3_, −0.2994)])
Γ_7_	([(𝕁_3_, 0.3647), (𝕁_6_, −0.0325), (𝕁_4_, −0.1893), (𝕁_5_, 0.4612)])	([(𝕁_3_, −0.3662), (𝕁_4_, 0.0793), (𝕁_3_, −0.1570), (𝕁_4_, 0.4322)])
	**Θ_3_**	**Θ_4_**
Γ_1_	([(𝕁_2_, −0.1757), (𝕁_3_, −0.1172), (𝕁_3_, −0.2714), (𝕁_4_, −0.1202)])	([(𝕁_3_, −0.3024), (𝕁_4_, −0.0000), (𝕁_2_, 0.1969), (𝕁_4_, −0.0812)])
Γ_2_	([(𝕁_3_, 0.2049), (𝕁_6_, −0.3070), (𝕁_2_, 0.4422), (𝕁_5_, −0.2384)])	([(𝕁_4_, −0.3978), (𝕁_5_, −0.4285), (𝕁_3_, −0.1119), (𝕁_5_, −0.4050)])
Γ_3_	([(𝕁_3_, 0.2641), (𝕁_4_, 0.3105), (𝕁_3_, −0.3141), (𝕁_4_, 0.1909)])	([(𝕁_3_, 0.4905), (𝕁_4_, 0.4822), (𝕁_2_, −0.0625), (𝕁_5_, −0.0135)])
Γ_4_	([(𝕁_3_, 0.1986), (𝕁_5_, −0.1912), (𝕁_2_, −0.2965), (𝕁_4_, −0.2675)])	([(𝕁_2_, 0.4242), (𝕁_4_, −0.1905), (𝕁_1_, 0.4557), (𝕁_3_, 0.3744)])
Γ_5_	([(𝕁_2_, 0.4605), (𝕁_3_, 0.3650), (𝕁_3_, −0.3970), (𝕁_4_, 0.2174)])	([(𝕁_4_, 0.4766), (𝕁_6_, 0.0546), (𝕁_2_, −0.1293), (𝕁_4_, 0.3781)])
Γ_6_	([(𝕁_4_, 0.3023), (𝕁_5_, 0.2651), (𝕁_1_, 0.4246), (𝕁_3_, −0.4386)])	([(𝕁_2_, 0.3291), (𝕁_4_, −0.1286), (𝕁_2_, 0.1738), (𝕁_4_, −0.2209)])
Γ_7_	([(𝕁_4_, 0.1250), (𝕁_5_, 0.0534), (𝕁_3_, −0.3687), (𝕁_4_, 0.2302)])	([(𝕁_3_, 0.1728), (𝕁_4_, 0.2082), (𝕁_2_, 0.2934), (𝕁_4_, −0.0911)])

**Table 9:** The evaluated ${𝕎\mathbb{N}}_{ı}$_*ȷ*_ matrix with 2TLIV*q*-ROFNs.

	**Θ_1_**	**Θ_2_**
Γ_1_	([(𝕁_2_, 0.0239), (𝕁_3_, −0.2288), (𝕁_6_, −0.2854), (𝕁_7_, −0.3455)])	([(𝕁_2_, 0.2582), (𝕁_3_, 0.0326), (𝕁_6_, −0.0812), (𝕁_7_, −0.3127)])
Γ_2_	([(𝕁_2_, −0.1370), (𝕁_3_, 0.2192), (𝕁_6_, 0.0122), (𝕁_7_, −0.3316)])	([(𝕁_2_, 0.4560), (𝕁_4_, −0.4154), (𝕁_5_, 0.4670), (𝕁_6_, 0.3916)])
Γ_3_	([(𝕁_3_, 0.2258), (𝕁_5_, −0.3595), (𝕁_6_, −0.1587), (𝕁_7_, −0.4484)])	([(𝕁_2_, −0.4768), (𝕁_3_, 0.2681), (𝕁_6_, 0.0432), (𝕁_7_, −0.1542)])
Γ_4_	([(𝕁_3_, −0.3130), (𝕁_4_, −0.2707), (𝕁_6_, −0.4181), (𝕁_7_, −0.4222)])	([(𝕁_2_, 0.1709), (𝕁_4_, −0.1247), (𝕁_6_, 0.3928), (𝕁_7_, −0.0296)])
Γ_5_	([(𝕁_2_, −0.0825), (𝕁_3_, −0.3379), (𝕁_6_, 0.4782), (𝕁_7_, 0.1644)])	([(𝕁_2_, 0.4395), (𝕁_4_, 0.0645), (𝕁_5_, 0.2703), (𝕁_6_, 0.2699)])
Γ_6_	([(𝕁_2_, 0.3023), (𝕁_3_, 0.0941), (𝕁_6_, −0.1131), (𝕁_7_, −0.2328)])	([(𝕁_1_, 0.2797), (𝕁_3_, −0.3394), (𝕁_5_, 0.3804), (𝕁_6_, 0.0761)])
Γ_7_	([(𝕁_3_, −0.3211), (𝕁_5_, −0.1954), (𝕁_7_, −0.3746), (𝕁_7_, 0.2601)])	([(𝕁_2_, 0.0952), (𝕁_3_, 0.2484), (𝕁_6_, 0.1557), (𝕁_7_, −0.1115)])
	**Θ_3_**	**Θ_4_**
Γ_1_	([(𝕁_1_, 0.4460), (𝕁_2_, 0.2854), (𝕁_6_, 0.1268), (𝕁_7_, −0.3143)])	([(𝕁_2_, 0.1333), (𝕁_3_, 0.1662), (𝕁_6_, −0.1675), (𝕁_7_, −0.2809)])
Γ_2_	([(𝕁_3_, −0.4590), (𝕁_5_, −0.4486), (𝕁_6_, −0.0394), (𝕁_7_, 0.0341)])	([(𝕁_3_, −0.1500), (𝕁_4_, −0.3770), (𝕁_6_, 0.2360), (𝕁_7_, −0.0143)])
Γ_3_	([(𝕁_3_, −0.4120), (𝕁_3_, 0.4220), (𝕁_6_, 0.1029), (𝕁_7_, −0.1852)])	([(𝕁_3_, −0.2386), (𝕁_4_, −0.4486), (𝕁_6_, −0.3439), (𝕁_7_, 0.1268)])
Γ_4_	([(𝕁_3_, −0.4640), (𝕁_4_, −0.1768), (𝕁_5_, 0.4512), (𝕁_7_, −0.3781)])	([(𝕁_2_, −0.0829), (𝕁_3_, 0.0147), (𝕁_5_, 0.2742), (𝕁_6_, 0.4778)])
Γ_5_	([(𝕁_2_, −0.0496), (𝕁_3_, −0.3319), (𝕁_6_, 0.0557), (𝕁_7_, −0.1745)])	([(𝕁_4_, −0.4531), (𝕁_5_, −0.1504), (𝕁_6_, −0.3922), (𝕁_7_, −0.0963)])
Γ_6_	([(𝕁_3_, 0.4154), (𝕁_4_, 0.1953), (𝕁_5_, 0.2148), (𝕁_6_, 0.0315)])	([(𝕁_2_, −0.1582), (𝕁_3_, 0.0638), (𝕁_6_, −0.1825), (𝕁_7_, −0.3403)])
Γ_7_	([(𝕁_3_, 0.2735), (𝕁_4_, 0.0219), (𝕁_6_, 0.0719), (𝕁_7_, −0.1694)])	([(𝕁_3_, −0.4905), (𝕁_3_, 0.3322), (𝕁_6_, −0.1058), (𝕁_7_, −0.2851)])

**Table 10:** Score functions of weighted normalized matrix.

	**Θ_1_**	**Θ_2_**	**Θ_3_**	**Θ_4_**
Γ_1_	0.3845	0.3746	0.3645	0.3758
Γ_2_	0.3722	0.4118	0.3504	0.3358
Γ_3_	0.3973	0.3566	0.3570	0.3462
Γ_4_	0.3968	0.3289	0.3978	0.4098
Γ_5_	0.3009	0.4261	0.3569	0.3814
Γ_6_	0.3697	0.4292	0.4416	0.3807
Γ_7_	0.2918	0.3474	0.3606	0.3741

**Table 11:** The evaluated ***G*** matrix with 2TLIV*q*-ROFNs.

***g*_ȷ_**	**The 2TLIV*q*-ROFNs for *g*_ȷ_**	** $F\left(g_{ȷ}\right)$ **
***g***_1_	([(𝕁_2_, 0.3420), (𝕁_3_, 0.4742)], [(𝕁_6_, 0.0941), (𝕁_7_, −0.1420)])	0.3538
***g***_2_	([(𝕁_2_, −0.0185), (𝕁_3_, 0.3601)], [(𝕁_6_, −0.1142), (𝕁_7_, −0.3531)])	0.3795
***g***_3_	([(𝕁_2_, 0.4473), (𝕁_3_, 0.4756)], [(𝕁_6_, −0.0924), (𝕁_7_, −0.2633)])	0.3722
***g***_4_	([(𝕁_2_, 0.4486), (𝕁_3_, 0.4719)], [(𝕁_6_, −0.2016), (𝕁_7_, −0.1748)])	0.3692

**Table 12:** Evaluated 2TLIV*q*-ROFN distance matrix.

**Alternative**	**Θ_1_**	**Θ_2_**	**Θ_1_**	**Θ_2_**
Γ_1_	0.0334	−0.0051	−0.0156	0.0108
Γ_2_	0.0199	0.0322	−0.0355	−0.0351
Γ_3_	0.0435	−0.0230	−0.0154	−0.0340
Γ_4_	0.0431	−0.0545	0.0256	0.0428
Γ_5_	−0.0528	0.0466	−0.0153	0.0259
Γ_6_	0.0176	0.0519	0.0694	0.0150
Γ_7_	−0.0825	−0.0322	−0.0183	0.0131

Consequently, using the result of $S_{ı}$, alternatives $\Gamma_{ı}=(ı=1,2,\ldots ,7)$) can be ordered accordingly. The alternative with the greatest $S_{ı}$ value is considered optimal. Following is the order of alternatives:


$$\Gamma_{6}>\Gamma_{4}>\Gamma_{1}>\Gamma_{5}>\Gamma_{2}>\Gamma_{3}>\Gamma_{7}$$

As a result, Γ_6_ is the most effective treatment for breast cancer.

### 6.3 Analyzing parameter effect on rankings

**Figure 2:**
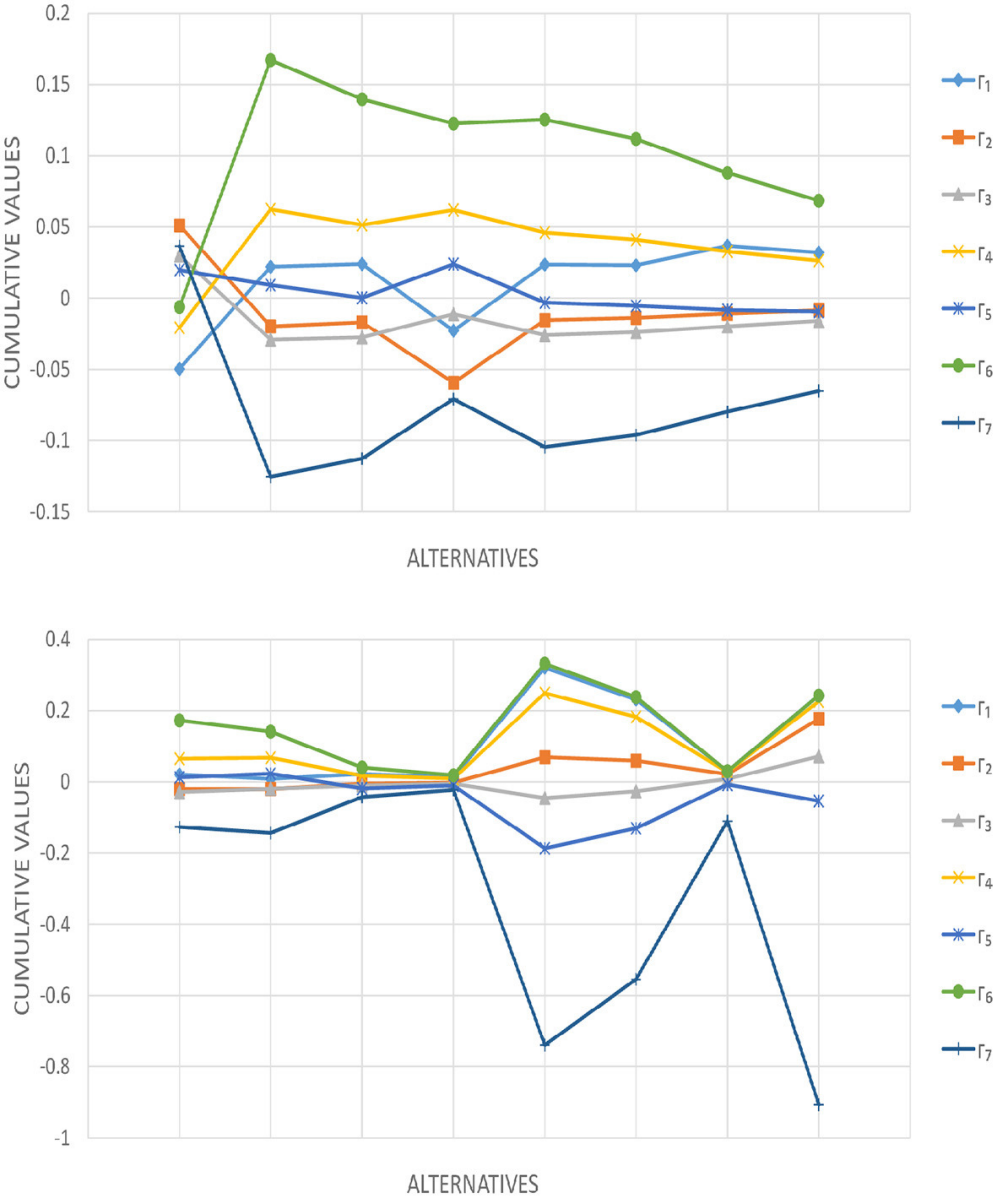
Cumulative values employing the 2TLIV*q*-ROFWA operator as depicted in Table 13.

Cumulative values and rankings based on various *q* values in the 2TLIV*q*-ROFWA operator are provided in Table 13. This research reveals how *q* affects the performance and ranking of seven alternatives. *q* is the parameter of the 2TLIV*q*-ROFS and always has a positive value because it represents a quantity that cannot be negative (i.e., negative root values can distort the results and make the information complex to evaluate). In the proposed model the values of parameter *q* are chosen randomly considering their condition to be positive to observe its impact on the final ranking. Trends show that choice outcomes are sensitive to *q*. Cumulative values for each alternative vary greatly when *q* changes. Alternative Γ_6_ regularly outperforms others, with cumulative values increasing with the *q* level. However, alternative Γ_7_ typically has the lowest results for different *q* values, indicating poor performance. This sensitivity to *q* emphasizes the need to carefully select this parameter in the decision-making process to match context and criterion. Further examination of the Table 13 shows that ranks change with *q*. Different *q* values significantly affect the ranking of alternatives, demonstrating the 2TLIV*q*-ROFWA operator's responsiveness to parameter changes. Notably, alternative Γ_6_ consistently ranks first for most *q* values, demonstrating its robust and beneficial performance under a variety of conditions. Conversely, alternative Γ_7_ frequently gets ranked lower, suggesting its inferior performance in most situations. To make educated judgments using the 2TLIV*q*-ROFWA aggregation operator, the best *q* value must be carefully evaluated, taking into consideration the relationship between alternatives and preferences of DMs. To evaluate these rankings' stability under data fluctuations and uncertainties, robustness testing is advised. In summary, the *q* parameter significantly affects the outcomes utilizing the 2TLIV*q*-ROFWA operator operator, requiring sensitivity analysis, a deep understanding of decision-making implications, and robustness assessment to ensure reliable rankings under changing data conditions. The visual variation of parameters can be observed in Figure 2.

**Table 13:** Ranking outcomes with different *q* in the 2TLIV*q*-ROFWA operator.

***q***	**Γ_1_**	**Γ_2_**	**Γ_3_**	**Γ_4_**	**Γ_5_**	**Γ_6_**	**Γ_7_**	**Ranking**
1/2	−0.0496	0.0511	0.0299	−0.0207	0.0197	−0.0063	0.0366	Γ_2_ > Γ_7_ > Γ_3_ > Γ_5_ > Γ_6_ > Γ_4_ > Γ_1_
5	0.0221	−0.0197	−0.0291	0.0624	0.0093	0.1672	−0.1253	Γ_6_ > Γ_4_ > Γ_1_ > Γ_5_ > Γ_2_ > Γ_3_ > Γ_7_
7	0.0239	−0.0169	−0.0276	0.0514	0.0002	0.1396	−0.1126	Γ_6_ > Γ_4_ > Γ_1_ > Γ_5_ > Γ_2_ > Γ_3_ > Γ_7_
3/2	−0.0227	−0.0593	−0.0111	0.0619	0.0239	0.1226	−0.0707	Γ_6_ > Γ_4_ > Γ_5_ > Γ_3_ > Γ_1_ > Γ_2_ > Γ_7_
8	0.0237	−0.0153	−0.0259	0.0460	−0.0030	0.1254	−0.1045	Γ_6_ > Γ_4_ > Γ_1_ > Γ_5_ > Γ_2_ > Γ_3_ > Γ_7_
9	0.0231	−0.0138	−0.0238	0.0411	−0.0054	0.1118	−0.0961	Γ_6_ > Γ_4_ > Γ_1_ > Γ_5_ > Γ_2_ > Γ_3_ > Γ_7_
11	0.0368	−0.0109	−0.0197	0.0328	−0.0083	0.0879	−0.0797	Γ_6_ > Γ_1_ > Γ_4_ > Γ_5_ > Γ_2_ > Γ_3_ > Γ_7_
13	0.0321	−0.0086	−0.0159	0.0262	−0.0094	0.0684	−0.0651	Γ_6_ > Γ_1_ > Γ_4_ > Γ_2_ > Γ_5_ > Γ_3_ > Γ_7_
9/2	0.0209	−0.0201	−0.0286	0.0648	0.0121	0.1730	−0.1269	Γ_6_ > Γ_4_ > Γ_1_ > Γ_5_ > Γ_2_ > Γ_3_ > Γ_7_
7/3	0.0083	−0.0198	−0.0195	0.0681	0.0231	0.1408	−0.1437	Γ_6_ > Γ_4_ > Γ_5_ > Γ_1_ > Γ_3_ > Γ_2_ > Γ_7_
17	0.0219	−0.0052	−0.0102	0.0170	−0.0189	0.0408	−0.0426	Γ_6_ > Γ_1_ > Γ_4_ > Γ_2_ > Γ_3_ > Γ_5_ > Γ_7_
23	0.0122	−0.0024	−0.0051	0.0090	−0.0101	0.0188	−0.0223	Γ_6_ > Γ_1_ > Γ_4_ > Γ_2_ > Γ_3_ > Γ_5_ > Γ_7_
57	0.3204	0.0694	−0.0453	0.2493	−0.1864	0.3321	−0.7393	Γ_6_ > Γ_1_ > Γ_4_ > Γ_2_ > Γ_3_ > Γ_5_ > Γ_7_
60	0.2312	0.0596	−0.0264	0.1825	−0.1307	0.2378	−0.5541	Γ_6_ > Γ_1_ > Γ_4_ > Γ_2_ > Γ_3_ > Γ_5_ > Γ_7_
101	0.0298	0.0213	0.0082	0.0277	−0.0072	0.0298	−0.1097	Γ_6_ > Γ_1_ > Γ_4_ > Γ_2_ > Γ_3_ > Γ_5_ > Γ_7_
103	0.2425	0.1766	0.0714	0.2261	−0.0536	0.2425	−0.9058	Γ_6_ > Γ_1_ > Γ_4_ > Γ_2_ > Γ_3_ > Γ_5_ > Γ_7_

### 6.4 Comparative analysis

**Figure 3:**
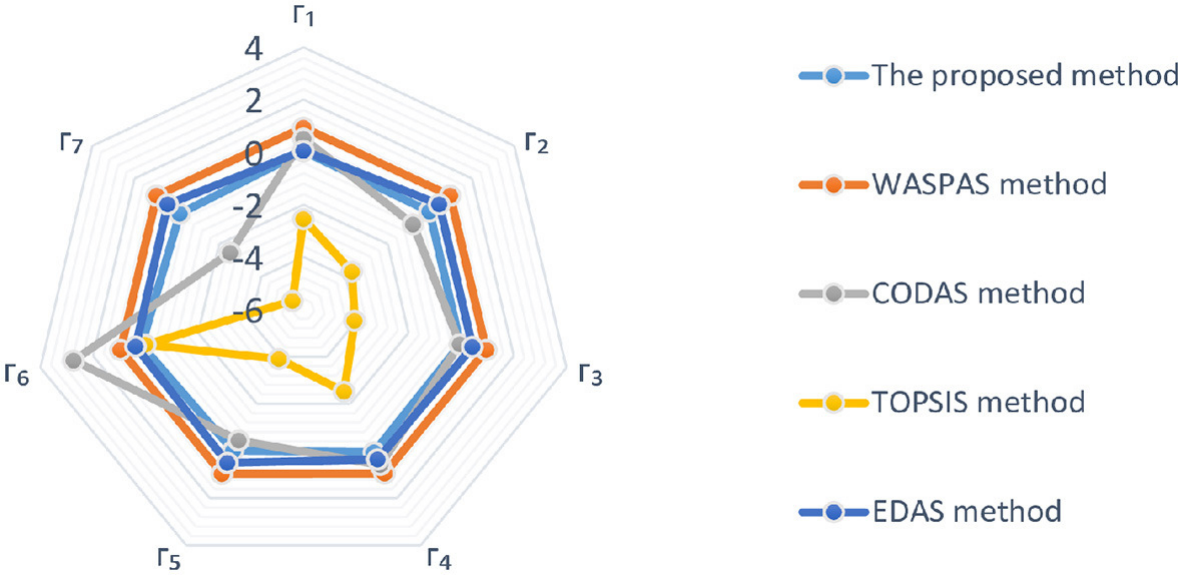
Cumulative values employing different MAGDM methods as depicted in Table 14.

Comparative analysis between the proposed research and the existing literature is the objective of this subsection, with the intent of highlighting the benefits of the new proposed study in relation to the existing theories. In this study, we present a case study and address it using our proposed method in addition to a large number of MAGDM techniques already in use. In response to a variety of practical decision problems, we propose a novel decision strategy based on multiple theories, known as the 2TLIV*q*-ROFS. In the context of risk decision-making complexities, many existing methodologies must incorporate the psychological dynamics and behavioral aspects of DMs throughout information synthesis and choice analysis. Tables 14, 15 display the 2TLIV*q*-ROFWA operator's comparative analysis of breast cancer treatment selection utilizing different MAGDM methods. In Table 14, the cumulative values for various treatment options are displayed, with each column representing a unique MAGDM method. Notably, alternative Γ_6_ consistently receives the highest cumulative value, whereas the rankings of the remaining alternatives differ across methods. For instance, the proposed method, the CODAS method (Keshavarz Ghorabaee et al., 2016), and the TOPSIS method (Hwang and Yoon, 1981) rank the same alternative as the best, whereas EDAS method (Keshavarz Ghorabaee et al., 2015) and the WASPAS method (Chakraborty and Zavadskas, 2014) produce distinct results, highlighting the significance of the chosen method for treatment selection. Table 15 provides a ranking of each MAGDM method's results to further illustrate these distinctions. According to each technique, the optimal solution is determined. Γ_6_ is ranked highest in three methodologies, highlighting its potential as a preferred treatment option. Other alternatives, however, are ranked significantly differently. The EDAS method and WASPAS method distinguish themselves by selecting Γ_3_ and Γ_5_ as the best options, respectively, highlighting the method's distinctive criteria and decision-making priorities. This comparative analysis emphasizes the impact of the selected MAGDM method on the ranking and selection of breast cancer treatment alternatives. It stresses the significance of understanding the specific criteria and preferences of each method when making decisions in the complex realm of breast cancer treatment. When using MAGDM techniques, researchers and healthcare professionals should carefully consider these variations to ensure that each patient receives the optimal treatment for his or her specific requirements. The visual depiction of comparative analysis results is given in Figure 3.

**Table 14:** Comparison with different MAGDM methods by the 2TLIV*q*-ROFWA operator.

	**Cumulative values by**	**Cumulative values by**	**Cumulative values by**	**Cumulative values by**	**Cumulative values by**
**Alternative**	**the proposed method**	**the WASPAS method (Chakraborty and Zavadskas, 2014)**	**the CODAS method (Keshavarz Ghorabaee et al., 2016)**	**the TOPSIS method (Hwang and Yoon, 1981)**	**the EDAS method (Keshavarz Ghorabaee et al., 2015)**
Γ_1_	0.0342	0.9385	0.5136	−2.5500	0.0549
Γ_2_	−0.0196	0.9551	−0.7960	−3.7053	0.4369
Γ_3_	−0.0316	0.9552	−0.0520	−4.0492	0.4314
Γ_4_	0.0451	0.9512	0.5868	−2.5172	0.3564
Γ_5_	−0.0079	0.9585	−0.4552	−3.9046	0.5000
Γ_6_	0.1594	0.9526	2.7360	0.0000	0.3755
Γ_7_	−0.1366	0.9548	−2.5332	−5.4905	0.4331

**Table 15:** Ranking results of comparative analysis.

**MAGDM methods**	**Ranking results**	**The best alternative**
The proposed method	Γ_6_ > Γ_4_ > Γ_1_ > Γ_5_ > Γ_2_ > Γ_3_ > Γ_7_	Γ_6_
WASPAS	Γ_5_ > Γ_3_ > Γ_2_ > Γ_7_ > Γ_6_ > Γ_4_ > Γ_1_	Γ_5_
CODAS	Γ_6_ > Γ_1_ > Γ_4_ > Γ_3_ > Γ_2_ > Γ_5_ > Γ_7_	Γ_6_
TOPSIS	Γ_6_ > Γ_1_ > Γ_4_ > Γ_2_ > Γ_5_ > Γ_3_ > Γ_7_	Γ_6_
EDAS	Γ_3_ > Γ_6_ > Γ_2_ > Γ_5_ > Γ_4_ > Γ_7_ > Γ_1_	Γ_3_

## 7 Conclusions

Many women worldwide suffer from deadly breast cancer now-a-days. Choosing the optimal course of treatment for each patient can be quite challenging due to the numerous variables and individual preferences at play. To address this complexity, treatment selection for breast cancer can be regarded as a MAGDM problem, in which a group of doctors and DMs evaluate and rank numerous options based on numerous attributes. Utilizing MAGDM techniques can enhance the effectiveness and quality of breast cancer treatment selection decisions. In this study, we introduced the concept of a 2TLIV*q*-ROFS to assist DMs in handling ambiguous information and express their evaluations in a broader domain. To accomplish this, we proposed a collection of novel aggregation operators, such as the 2TLIV*q*-ROFWA and 2TLIV*q*-ROFWJ operators. In addition, by employing 2TLIV*q*-ROF information, we expanded the MABAC method to address the MAGDM issue. We conducted a case study of breast cancer treatment selection to demonstrate the efficacy and viability of the proposed methodology. In complex decision-making scenarios, the computational outcomes indicate that our MAGDM model is capable of handling ambiguity and uniqueness. A sensitivity analysis is also performed to ascertain whether the effects of the option are affected by varying the parameter *q*. DMs can gain insight into how this parameter influences results by observing its impact, and can then make informed adjustments as required. The results from the comparison with existing studies indicate that our method can handle the complexity and uncertainty associated with breast cancer treatment selection while still providing a positive outcome for both patients and medical professionals. Although, the proposed approach presents potential benefits in addressing the complexity of treatment selection by incorporating various attributes and preferences, some of its limitations are existed. There is no discussion on the ethical implications and potential biases associated with the use of MAGDM techniques in healthcare decision-making. The proposed approach does not address the challenges or limitations of integrating machine learning and artificial intelligence, including data privacy concerns and algorithmic biases. Future research opportunities may include integrating technologies such as machine learning and artificial intelligence into the MAGDM framework for selecting breast cancer treatments. This integration will increase the precision and efficacy of decision-making processes and contribute to improved patient outcomes. In various areas of healthcare decision-making, the proposed strategy will be extended to more informed choices and can benefit more individuals by discussing ethical implications and potential biases.

## Data availability statement

The original contributions presented in the study are included in the article/Supplementary material, further inquiries can be directed to the corresponding author.

## Author contributions

MR: Writing – review & editing, Writing – original draft, Visualization, Validation, Supervision, Software, Resources, Project administration, Methodology, Investigation, Funding acquisition, Formal analysis, Data curation, Conceptualization. AM: Writing – review & editing, Writing – original draft, Visualization, Validation, Supervision, Software, Resources, Project administration, Methodology, Investigation, Funding acquisition, Formal analysis, Data curation, Conceptualization. ANM: Writing – review & editing, Writing – original draft, Project administration, Funding acquisition. IB: Writing – review & editing, Writing – original draft. ZA: Writing – review & editing, Writing – original draft. ZF: Writing – review & editing, Writing – original draft.
