# Supplementary figures and images for: Enhancing breast cancer treatment selection through 2TLIVq-ROFS-based multi-attribute group decision making
Source: Front Artif Intell. 2024 Jun 3;7:1402719. doi: 10.3389/frai.2024.1402719 (PMC11180902; doi:10.3389/frai.2024.1402719)

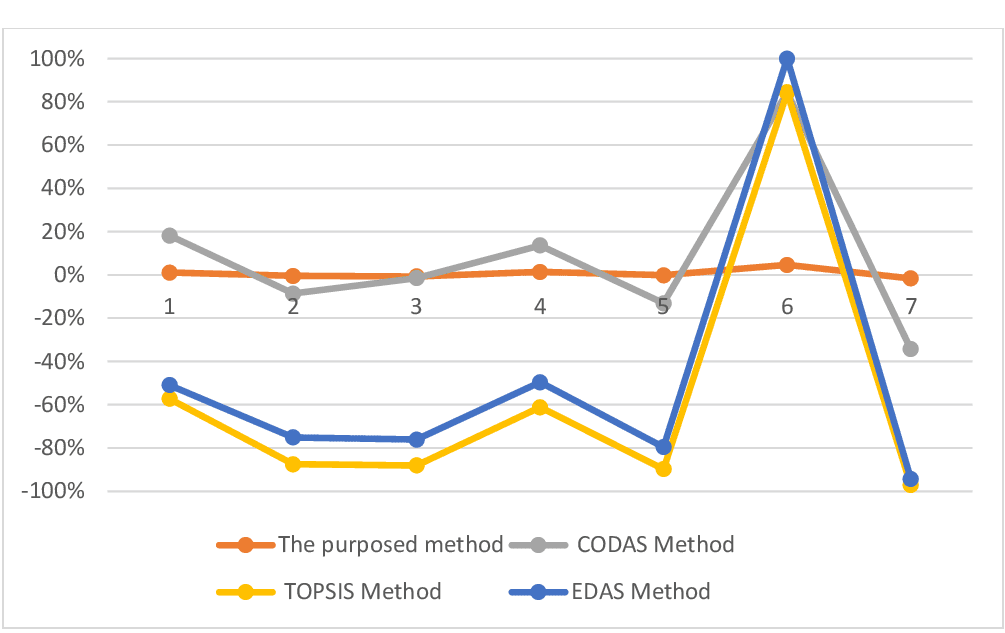

Supplement: Supplementary file 1 [file Image_1.PNG]

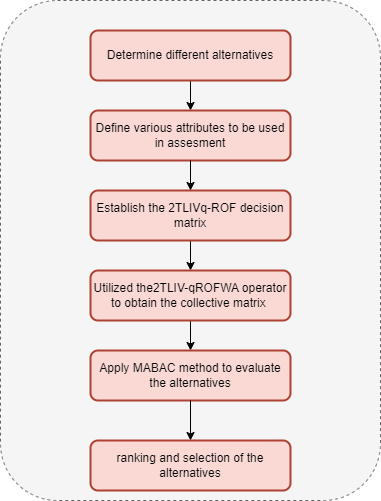

Supplement: Supplementary file 2 [file Image_2.PNG]
